# AR to GR switch modulates differential TDO2-Kyn-AhR signalling to promote the survival and recurrence of treatment-induced dormant cells in prostate cancer

**DOI:** 10.1038/s41421-025-00817-w

**Published:** 2025-08-05

**Authors:** Sangsang Li, Yifan Zhang, Maoxing Luo, Weiwei Zhou, Yitong Chen, Dinglan Wu, Qiang Wei, Yan Chang, Hailiang Hu

**Affiliations:** 1 Department of Biochemistry, SUSTech Homeostatic Medicine Institute, School of Medicine, Southern University of Science and Technology, Shenzhen, China; 2 Department of Urology, Nanfang Hospital, Southern Medical University, Guangdong, Guangzhou China; 3 Shenzhen Key Laboratory of Viral Oncology, Clinical Innovation and Research Center (CIRC), Shenzhen Hospital of Southern Medical University, Shenzhen, China; 4 Institute of Clinical Pharmacology, Anhui Medical University, Key Laboratory of Anti-Inflammatory and Immune Medicine, Ministry of Education, Hefei, China; 5 Laboratory Animal Center, Anhui Medical University, Hefei, China; 6 Key University Laboratory of Metabolism and Health of Guangdong, Southern University of Science and Technology, Shenzhen, China

**Keywords:** Cancer metabolism, Cell signalling

## Abstract

Cancer cells can be induced to dormancy initially by specific cancer therapies, but can be reactivated for subsequent relapse as therapy-resistant cancer cells. Although the treatment-induced dormancy-to-reactivation switch is an important process in tumour spread and recurrence, little is known about the underlying molecular mechanisms, particularly the metabolic underpinnings. In this study, we demonstrated that the tryptophan catabolism-related tryptophan 2,3-dioxygenase (TDO2) -kynurenine (Kyn) -aryl hydrocarbon receptor (AhR) signalling axis was responsible for both sustaining the survival of dormant prostate cancer cells induced by androgen deprivation therapy (ADT) and promoting the reactivation of dormant cells and their recurrent outgrowth, which facilitated the development of therapeutic resistance by allowing the dormancy-to-reactivation switch. Mechanistically, we found that ADT upregulated the expression of TDO2 to produce Kyn, which activated AhR and maintained the survival of ADT-induced dormant cells. Interestingly, the switch of transcription factors from the androgen receptor (AR) to the glucocorticoid receptor (GR) modulated the persistent expression of TDO2 and promoted the reactivation of dormant cells through the same TDO2-Kyn-AhR signalling axis. Additionally, tumour recurrence following ADT was delayed by pharmacological suppression of TDO2-Kyn-AhR signalling with a TDO2 inhibitor or an AhR inhibitor. In summary, we describe a signalling circuit mediated by tryptophan metabolism for regulating tumour cell dormancy and recurrence and propose TDO2 as a new target for the treatment of androgen-sensitive prostate cancer patients in combination with ADT.

## Introduction

Cancer recurrence after the development of therapy resistance usually results in the failure of cancer treatment^1^. Tumour dormancy has been proposed to explain the latency period between the initial effective treatment and later cancer recurrence, which includes two mechanisms—tumour mass dormancy and cellular dormancy^2^. Tumour mass dormancy results from the interaction between tumour cells and the cells of their environment, which allows tumours to reach a balance between cell proliferation and cell death, whereas cellular dormancy occurs when tumour cells enter a quiescent state but retain the ability to regrow to cause tumour recurrence later^3^. Given that dormant tumour cells are resistant to many types of cancer therapy, such as chemotherapy or targeted therapy^4,5^, understanding the underlying mechanisms of therapy-induced tumour dormancy, which may help in the development of new treatment strategies for targeting dormant tumour cells, is imperative.

Prostate cancer has been used as a disease model for studying tumour dormancy, as prostate tumours maintain a dormant state for a long period of time locally or distantly before they progress to overt tumours^6,7^. Androgen deprivation therapy (ADT) is a standard therapy for androgen-sensitive prostate cancer patients that leads to a dormancy period for prostate tumours that is recognised clinically as a minimal residual disease^8,9^. However, the majority of these tumours will escape dormancy and eventually recur as castration-resistant prostate cancer, thus providing clinical relevance for studying ADT-induced tumour dormancy. Most recently, Dong et al. established a panel of hormonally sensitive prostate cancer patient-derived xenograft (PDX) models to study ADT-induced dormancy in vivo and revealed two ADT-induced prostate cancer dormancy subtypes differing in morphology, gene expression and biological recurrence^10^. However, the molecular mechanism, especially metabolic alterations, involved in ADT-induced prostate tumour dormancy and recurrence remains largely unexplored.

Tryptophan is an essential amino acid that can function as a precursor for multiple signalling metabolites^11^. Altered tryptophan metabolism has been reported in many solid tumours^12–15^, and 95% of tryptophan catabolism in animal cells occurs through the kynurenine (Kyn) pathway, which leads to the synthesis of NAD+ along with the production of the signalling metabolite Kyn^16,17^. Indoleamine 2,3-dioxygenase 1 (IDO1), indoleamine 2,3-dioxygenase 2 (IDO2) and tryptophan 2,3-dioxygenase (TDO2) are three reported rate-limiting enzymes for the first step of the Kyn pathway, and their expression patterns differ: IDO1 is expressed in many tissues and can be induced by interferon γ (IFN-γ), IDO2 is expressed primarily in the immune system, and TDO2 is expressed predominantly in the liver^18^. The signalling metabolite Kyn has been shown to serve as an endogenous ligand to activate aryl hydrocarbon receptor (AhR)^19^, a transcription factor that can induce the transcription of a subset of genes to enable cells to sense and respond to xenobiotic compounds^20^. The Kyn-AhR axis has been shown to enhance the malignant phenotype of cancer cells^21–23^ but suppress immune surveillance^24–27^. More specifically, the IDO1-Kyn-AhR pathway has been shown to regulate the immune dormancy of tumour-repopulating cells treated with IFN-γ^28^, whereas the TDO2-Kyn-AhR axis has been demonstrated to play a critical role in protecting against anoikis in suspended breast cancer cells^12^ and in increasing glycolysis to drive APC-deficient colorectal cancer growth and recruiting macrophages into the tumour microenvironment to suppress immune surveillance^22^.

In this study, we characterized TDO2-mediated tryptophan catabolism as a functional regulator of cellular dormancy during ADT-induced prostate cancer dormancy and recurrence. Specifically, we demonstrated that TDO2 increased the survival of dormant prostate cancer cells through a TDO2-Kyn-AhR signalling axis, which in turn promoted tumour dormancy. Additionally, the switch in transcription factors from androgen receptor (AR) to glucocorticoid receptor (GR) modulated the persistent expression of TDO2, and the same TDO2-Kyn-AhR signalling pathway promoted the aggressive proliferation of prostate cancer cells in the ADT-resistant cell stage. Consequently, our results suggest that targeting TDO2 is a promising strategy for eliminating dormant prostate cancer and preventing tumour recurrence and represents a new therapeutic avenue for targeting recurrent castration-resistant prostate cancer.

## Results

### ADT induces TDO2 upregulation in androgen-dependent prostate cancer

**Fig. 1:**
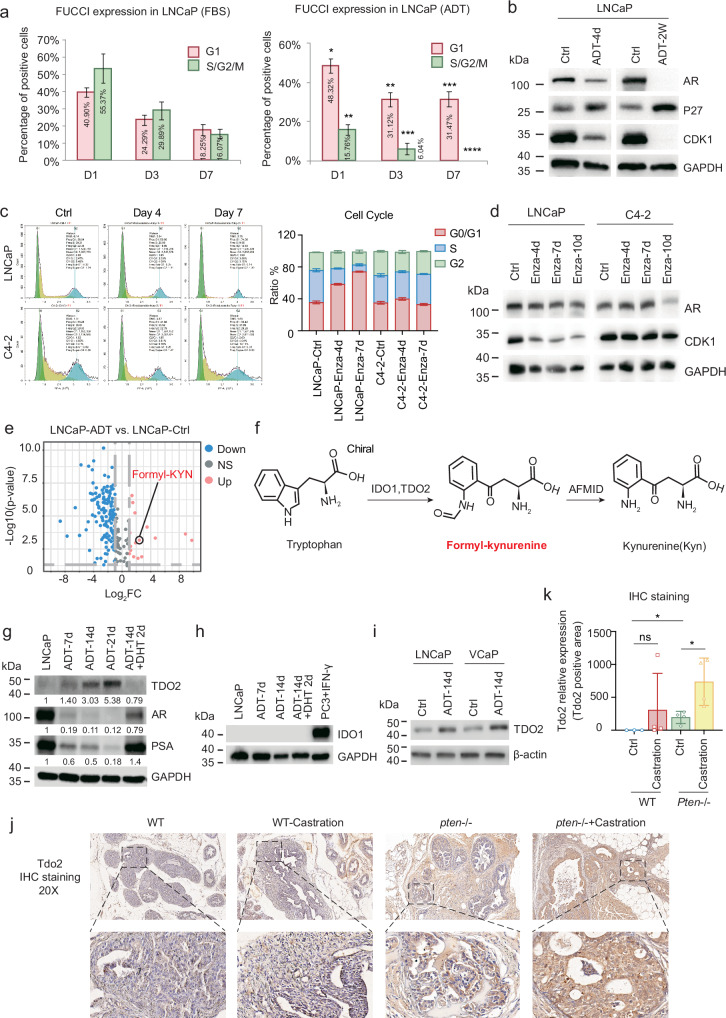
ADT induces TDO2 upregulation in androgen-dependent prostate cancer. **a** FUCCI system was used to show that ADT for 7 days led most cells to enter the G1 phase of the cell cycle. **b** The protein expression of AR, P27 and CDK1 in LNCaP cells treated with ADT for 4 days or 2 weeks was assessed by Western blotting. **c** Cell cycle distribution of LNCaP and C4-2 cells treated with enzalutamide for 4 and 7 days. **d** The protein expression of AR and CDK1 in LNCaP and C4-2 cells after 4, 7 and 10 days of enzalutamide treatment was examined by western blotting assay. **e** Volcano map (day 14) showing that ADT significantly decreased the levels of most metabolites but increased the levels of a few metabolites. **f** Formyl-kynurenine is the precursor of Kyn, the first product catalysed by IDO1/2 and/or TDO2 during tryptophan catabolism. **g** The protein expression of TDO2, AR and PSA in LNCaP cell lines was examined by western blotting assay at different time points after ADT treatment (days 7, 14, and 21) and DHT supplementation. **h** The protein expression of IDO1 in LNCaP cell lines was assessed at different time points after ADT (days 7 and 14) and DHT supplementation. PC3 cells treated with IFN-γserved as a positive control for IDO1 protein expression. **i** TDO2 protein expression in LNCaP and VCaP cell lines subjected to ADT for 14 days was examined by western blotting assay. **j**, **k** Immunohistochemical staining for TDO2 was performed on prostate tissues from wild-type and *Pten*^*−/−*^ cKO mice, either castrated or non-castrated (n = 4). *P < 0.05; **P < 0.01; ***P < 0.001; ****P < 0.0001.

A notable characteristic of cellular dormancy is quiescence. LNCaP cells, an androgen-dependent prostate cancer cell line, exhibit an increased quiescent phenotype when cultured in charcoal-stripped serum (CSS) medium (which mimics ADT in vitro), and this quiescence is reversible when ADT is removed^29^. We used a fluorescence ubiquitin cell cycle indicator (FUCCI) system to further verify this observation by determining whether ADT causes the majority of cells to enter the G1 phase of the cell cycle. Over the course of treatment, as shown in Fig. 1a and Supplementary Fig. S1a, ADT increased the proportion of LNCaP cells in the G1 phase, as determined by mKO2-hCdt1 labelling. Additionally, cell cycle analysis revealed that as the duration of ADT increased, the percentage of LNCaP cells in the G1 phase increased (Supplementary Fig. S1b). ADT also resulted in an increase in the expression level of the cyclin-dependent kinase inhibitor P27 (also known as Kip1), a well-known cellular dormancy marker^30^ (Fig. 1b). These findings are in line with those of in vivo studies in the Gene Expression Omnibus (GEO) database, which revealed that *P27* mRNA levels were significantly elevated in *Pten* conditional knockout (*Pten*^−/−^ cKO) mice that underwent castration surgery (Supplementary Fig. S1c). After two weeks of ADT, the expression of cyclin-dependent kinase 1 (CDK1), a cell division regulatory protein necessary for entry into S phase and mitosis, was completely absent due to the overexpression of P27 (Fig. 1b). We administered enzalutamide, an androgen receptor inhibitor, to LNCaP and C4-2 cells to determine whether the drug treatment might also produce dormant cells. Although enzalutamide was cytotoxic to both cell types (Supplementary Fig. S1d), we found that enzalutamide administration resulted in cell cycle arrest at the G0/G1 phase and a notable decrease in CDK1 in LNCaP cells but not in C4-2 cells (Fig. 1c, d), suggesting that LNCaP cells are an ideal in vitro cell model for studying treatment (ADT or enzalutamide)-induced cellular dormancy. Moreover, three other clinically used anti-androgen agents (bicalutamide, apalutamide and darolutamide) were able to significantly arrest LNCaP cells at the G0/G1 phase (Supplementary Fig. S1e) and were all toxic to LNCaP cells (Supplementary Fig. S1f).

To investigate whether ADT induces metabolic changes, we used mass spectrometry to profile the metabolites of LNCaP cells cultured in normal FBS-containing media and charcoal-stripped FBS-containing media at days 1, 5, and 14^31^. As expected, ADT significantly reduced the levels of the majority of metabolites associated with energy while increasing the levels of a small number of metabolites (Fig. 1e; Supplementary Fig. S2a and Table S1). We previously demonstrated that the switch between glutamine, an ADT-induced upregulated metabolite, and its related glutaminase isoform drives prostate cancer progression and the development of resistance to hormone therapy^31^. Formyl-kynurenine, another ADT-induced upregulated metabolite, is the initial product of tryptophan catabolism, formed by the enzymatically catalysed oxidative cleavage of the tryptophan indole moiety and further converted to Kyn and then downstream metabolites in the subsequent steps^18^ (Fig. 1f; Supplementary Fig. S2b, c). Given that formyl-kynurenine was significantly upregulated by ADT, it is highly likely that ADT may induce IDO1 and/or TDO2 expression in prostate cancer (IDO2 has been reported to be expressed mainly in immune cells^32,33^). Interestingly, ADT upregulated TDO2 expression but not IDO1 expression in LNCaP cells (Fig. 1g, h; Supplementary Fig. S2d). Adding the androgen DHT abrogated the TDO2 upregulation (Fig. 1g; Supplementary Fig. S2d), suggesting that AR signalling may negatively regulate TDO2 expression. Additionally, ADT-induced TDO2 upregulation was also observed in another androgen-dependent prostate cancer cell line, VCaP (Fig. 1i), indicating that this phenomenon may be general to androgen-dependent prostate cancers. Indeed, we did not find TDO2 upregulation by enzalutamide treatment in androgen-independent C4-2 cells (Supplementary Fig. S2e).

To validate the ADT-induced TDO2 upregulation in vivo, we performed immunohistochemical (IHC) staining of prostate tissues from wild-type control and *Pten*^*−/−*^ cKO mice (Supplementary Fig. S3a)^34,35^ and found that TDO2 expression was significantly upregulated in *Pten*^*−/−*^ cKO mice compared with control mice and was further increased in castrated *Pten*^*−/−*^ cKO mice (Fig. 1j, k). This finding was in line with the RNA sequencing results from *Pten*^*−/−*^ cKO mice, in which *Pten*^*−/−*^ cKO mice subjected to castration surgery presented considerably elevated *Tdo2* mRNA levels, whereas control mice showed no changes with or without castration surgery (Supplementary Fig. S3b). Taken together, these findings indicate that a short duration of ADT (less than 3 weeks) resulted in the temporary dormancy of androgen-dependent prostate cancer cells and induced TDO2 upregulation in androgen-dependent prostate cancer.

### ADT-induced TDO2 upregulation prevents the death of dormant cells via the Kyn-AhR pathway

**Fig. 2:**
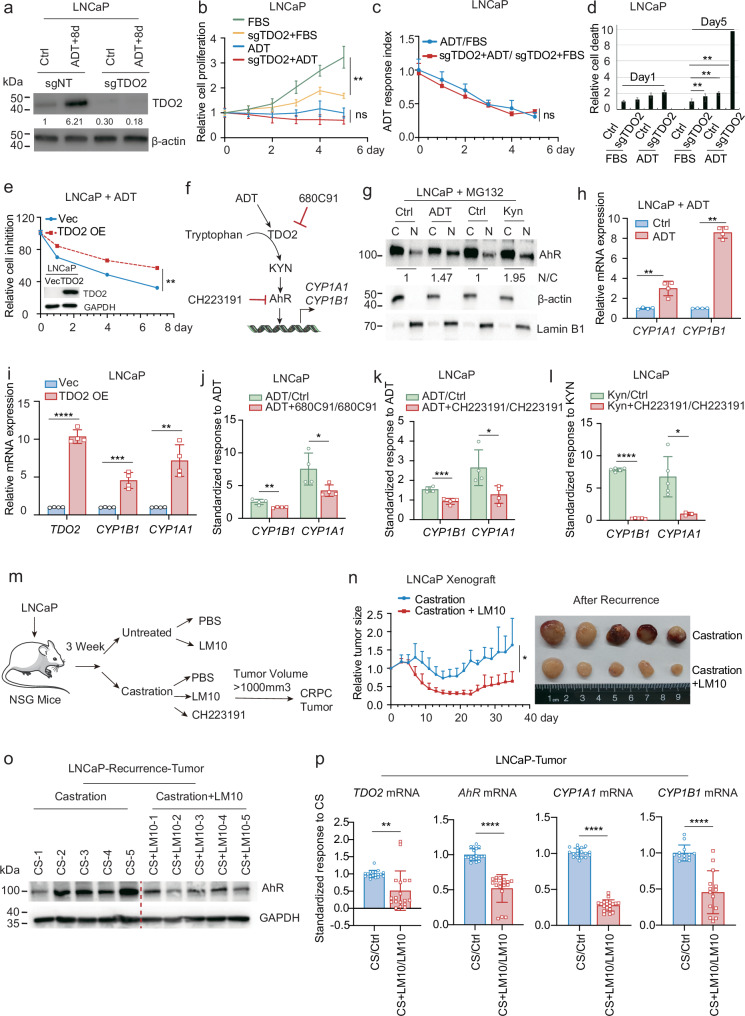
ADT-induced TDO2 upregulation prevents the death of dormant cells via the Kyn-AhR pathway. **a** CRISPR-Cas9 was used to knock out TDO2 in LNCaP cells, and western blotting assay was used to assess the protein expression of TDO2 under ADT. **b** Cell proliferation was tested on days 0, 1, 2, 3, 4, and 5 for LNCaP/Ctrl and LNCaP sgTDO2 cells with or without ADT. **c** The cell proliferation rate was normalized for LNCaP/Ctrl and LNCaP sgTDO2 cells. **d** Cell death was assessed on days 1 and 5 in LNCaP/Ctrl and LNCaP sgTDO2 cells with or without ADT. **e** LNCaP/Vec and LNCaP/TDO2 overexpression cells with or without ADT were tested for cell growth on days 0, 1, 4, and 7, and the cell numbers were normalised to show the ADT response. **f** Schematic diagram of the TDO2-Kyn-AhR pathway. **g** After ADT or Kyn treatment of LNCaP cells for 6 h, isolation of nuclear and cytoplasmic fractions was conducted, followed by WB analysis of the distribution of AhR in the cytoplasm and nucleus. **h** The mRNA levels of *CYP1A1* and *CYP1B1* were measured in LNCaP cells with or without ADT (48 h). **i** *CYP1A1* and *CYP1B1* mRNA levels were measured in LNCaP/Vec and LNCaP/TDO2 OE cells. **j** *CYP1A1* and *CYP1B1* mRNA levels were measured 48 h after the addition of the TDO2 inhibitor 680C91, ADT or both to LNCaP cells. The data were normalised to show the relative effect of 680C91 on ADT-induced AhR activation. **k** *CYP1A1* and *CYP1B1* mRNA levels were measured in LNCaP cells 24 h after the addition of the AhR inhibitor CH223191, ADT or their combination. The data were normalised to show the relative effect of CH223191 on ADT-induced AhR activation. **l** *CYP1A1* and *CYP1B1* mRNA levels were measured 24 h after the addition of the AhR inhibitor CH223191, Kyn or their combination to LNCaP cells. The data were normalized to show the relative rescue effect of Kyn on AhR activation. **m** Experimental model of LNCaP subcutaneous tumour formation in mice. **n** LNCaP cells were subcutaneously seeded into the axillary subcutaneous tissue of NSG mice and allowed to grow for 3 weeks. The mice were treated with a placebo or the TDO2 inhibitor LM10 after castration surgery (*n* = 5). **o** Protein was extracted from LNCaP xenograft tumours, and the expression of AhR was assessed by western blotting assay (*n* = 5). **p** RNA from LNCaP xenograft tumours was extracted, and the mRNA expression levels of *TDO2, AhR, CYP1A1* and *CYP1B1* were assessed by RT‒qPCR (*n* = 5). *P < 0.05; ** *P* < 0.01; *** *P* < 0.001; **** *P* < 0.0001.

To examine the cellular functions of ADT-induced TDO2 upregulation in dormant cells, we used CRISPR-Cas9 technology to knock out TDO2 in LNCaP cells and established a stable TDO2 knockout cell line in which TDO2 expression was no longer induced by ADT (Fig. 2a; Supplementary Fig. S4a). Despite growing slower than control cells in both the presence and absence of ADT, the TDO2 knockout cells presented a proliferative rate in response to ADT that did not change (Fig. 2b, c), suggesting that TDO2 knockout did not affect the proliferative capacity of cells in response to ADT. However, TDO2 knockout obviously increased ADT-induced cell death (Fig. 2d), suggesting that ADT-mediated TDO2 upregulation was necessary for cell survival rather than for cell proliferation. On the other hand, overexpressing TDO2 in LNCaP cells improved the resistance of cells to ADT (Fig. 2e; Supplementary Fig. S4b), indicating that TDO2 upregulation functioned to prevent prostate cancer cell death after ADT.

TDO2, like IDO1/2, is the first rate-limiting enzyme of the Kyn pathway and catalyzes the conversion of tryptophan to formyl-Kyn and then Kyn (Fig. 2f). Kyn can function as an endogenous ligand to activate AhR^19^, which leads to the transcription of prototypical genes, including drug metabolism enzyme-encoding genes such as cytochrome P450 family 1 members A1 (*CYP1A1*) and B1 (*CYP1B1*)^36^ (Fig. 2f). We conducted the following experiments to demonstrate that ADT-induced TDO2 upregulation activated the Kyn-AhR pathway. First, we performed a nucleocytoplasmic separation experiment to demonstrate that both ADT and Kyn could significantly increase the proportion of AhR in the nucleus (Fig. 2g), which was also verified by immunofluorescence staining experiments (Supplementary Fig. S5a). We subsequently measured the mRNA levels of *CYP1A1* and *CYP1B1* in LNCaP cells treated with or without ADT and found that ADT significantly increased the expression of *CYP1A1* and *CYP1B1* (Fig. 2h), suggesting that ADT could activate AhR. Second, as ADT upregulated TDO2 expression, which may catalyze tryptophan to produce more Kyn (Fig. 1f), the ADT-induced activation of AhR suggested that TDO2 may mediate this activation. TDO2 overexpression resulted in the upregulation of *CYP1A1* and *CYP1B1* mRNA expression in LNCaP cells (Fig. 2i), whereas TDO2 or AhR knockdown by siRNA abrogated ADT-induced *CYP1A1* mRNA upregulation (Supplementary Fig. S6a). The TDO2 inhibitor 680C91 or the AhR inhibitor CH223191 could also block ADT-induced AhR nuclear translocation (Supplementary Fig. S6b) and diminish the ADT-induced upregulation of *CYP1A1* and *CYP1B1* (Fig. 2j, k; Supplementary Fig. S6c, d), indicating that TDO2-mediated ADT-induced AhR activation. Finally, Kyn significantly increased the mRNA levels of *CYP1A1* and *CYP1B1* (Fig. 2l; Supplementary Fig. S6e), and the AhR inhibitor CH223191, rather than the TDO2 inhibitor, blocked Kyn-promoted AhR nuclear translocation (Supplementary Fig. S6f) and abrogated Kyn-induced increase in *CYP1A1* and *CYP1B1* (Fig. 2l; Supplementary Fig. S6e). Taken together, these results demonstrate that ADT activated AhR through TDO2 and Kyn.

Next, we wanted to determine whether the TDO2-Kyn-AhR pathway prevents ADT-induced cell death. Compared with ADT alone, the combination of ADT with the TDO2 inhibitor 680C91 or the AhR inhibitors CH223191 or BAY2416964 (another more potent AhR inhibitor) resulted in reduced cell survival (Supplementary Fig. S7a, b). Furthermore, Kyn supplementation restored the survival of LNCaP cells treated with ADT (Supplementary Fig. S7c) and further rescued the survival of cells treated with ADT and the TDO2 inhibitor 680C91 (Supplementary Fig. S7d), suggesting that Kyn mediated the protective function of ADT-induced cell death through TDO2. Further apoptosis analysis by flow cytometry revealed that the combination of ADT and the TDO2 inhibitor 680C91 or the AhR inhibitors CH223191 or BAY2416964 induced a greater degree of apoptosis than ADT alone (Supplementary Fig. S7e, f). This finding may suggest a potential treatment option for prostate cancer by inhibiting TDO2 or AhR together with ADT, as this may eradicate ADT-induced dormant cells. Similarly, Kyn significantly reduced apoptosis in LNCaP cells treated with the TDO2 inhibitor 680C91 and ADT (Supplementary Fig. S7g).

Kyn has been reported to inhibit ferroptosis via an AhR-independent cell-protective pathway^21^. To test whether Kyn can suppress ferroptosis through TDO2-Kyn-AhR signalling in response to ADT, we examined lipid peroxidation in LNCaP cells treated with or without ADT. Interestingly, ADT significantly induced lipid peroxidation in LNCaP cells, and compared with ADT alone, siRNA interference with TDO2 or AhR further increased the level of lipid peroxidation (Supplementary Fig. S8a, b). Similarly, Kyn abrogated the siTDO2- or TDO2 inhibitor 680C91-induced ferroptosis of LNCaP cells (Supplementary Fig. S8c, d), suggesting that Kyn partially inhibited ADT-induced cellular ferroptosis through the TDO2-Kyn-AhR signalling pathway.

Finally, we used an LNCaP xenograft mouse model to test the combination treatment in vivo and found that, compared with castration alone, the combination of TDO2i LM10 (which has demonstrated less toxicity than 680C91^30^) with castration further suppressed tumour growth and delayed tumour recurrence (Fig. 2m, n; Supplementary Fig. S9a, b). This was achieved by castrating NSG mice three weeks after LNCaP xenograft tumour implantation and then treating them with either a placebo or LM10. We extracted proteins and RNA from these xenograft tumours and found that LM10 significantly reduced the protein levels of AhR in castration-recurrent tumours (Fig. 2o). Compared with control LNCaP xenografts, LM10-treated xenografts also significantly reduced the mRNA levels of *TDO2*, *AhR*, *CYP1A1* and *CYP1B1* in castration-recurrent tumours (Fig. 2p; Supplementary Fig. S9c). Furthermore, compared with castration alone, combined treatment of CH223191 and castration resulted in greater inhibition of xenograft tumour growth (Supplementary Fig. S9d). Taken together, these findings indicate that ADT-induced upregulation of TDO2 prevented ADT-induced death of dormant prostate cancer cells and preserved drug tolerance, whereas TDO2 or AhR inhibition promoted ADT-induced death of dormant cells.

### Persistent TDO2 expression in CRPC also promotes tumour progression via the Kyn-AhR pathway

**Fig. 3:**
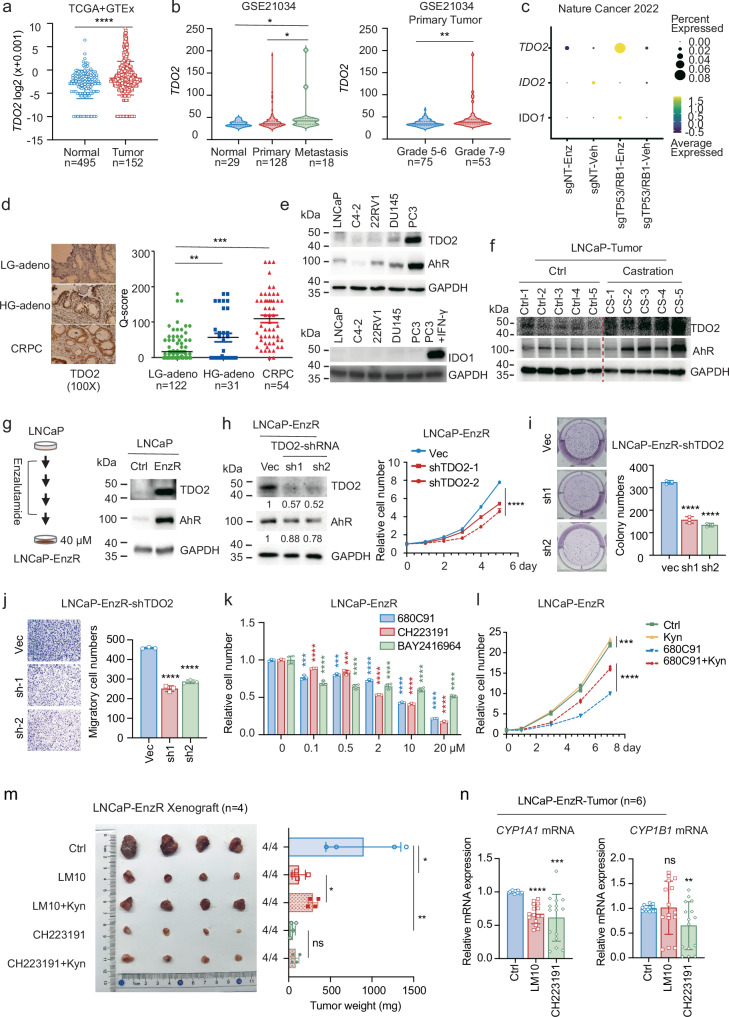
Persistent TDO2 expression in CRPC also promotes tumour progression and drug resistance via the Kyn-AhR pathway. **a** TDO2 mRNA levels were analyzed in normal prostate tissues (*n* = 495) and prostate cancer tissues (*n* = 152) from The Cancer Genome Atlas (TCGA) and the Genotype-Tissue Expression (GTEx) databases. **b** TDO2 mRNA expression levels in normal prostate tissues (*n* = 29), primary prostate cancer tissues (*n* = 128) and metastatic prostate cancer tissues from the GEO database (GSE21034) were analysed. The primary prostate cancer tissue was further analysed according to Gleason grade. **c** Analysis of TDO2 mRNA levels in CRPC cell lines from published single-cell sequencing results. **d** Immunohistochemical staining analysis was performed on the tissues of patients at low-grade (LG), high-grade (HG) and CRPC stages. The Q score was used to semi-quantify the TDO2 staining. **e** Western blotting was used to assess the protein expression levels of TDO2 and AhR in the prostate cancer cell lines LNCaP, C4-2, 22RV1, DU145 and PC3. **f** The protein expression levels of IDO1 in the prostate cancer cell lines LNCaP, C4-2, 22RV1, DU145 and PC3 were examined by Western blot, and PC3 + IFN-γ was used as the positive control for IDO1 expression. **g** An enzalutamide-resistant LNCaP cell line was established, and the protein levels of TDO2 and AhR were examined by western blotting assay. **h**–**j** TDO2 was knocked down by shRNA in LNCaP-EnzR cells, the protein levels of TDO2 and AhR were assessed (**h**), and cell proliferation, colony formation (**i**), and cell migration (**j**) were examined. **k** LNCaP-EnzR cells were treated with the TDO2 inhibitor 680C91 and the AhR inhibitor CH223191/BAY2416964 to assess cell inhibition. **l** The proliferation of LNCaP-EnzR cells treated with the TDO2 inhibitors 680C91 and 680C91+Kyn was examined on days 0, 1, 3, 5 and 7. **m** LNCaP-EnzR xenograft tumours were treated separately with a placebo, the TDO2 inhibitor LM10, the TDO2 inhibitor LM10 + Kyn, the AhR inhibitor CH223191, or the AhR inhibitor CH223191 + Kyn. The tumour weights were recorded (*n* = 4). **n** RNA from LNCaP-EnzR xenograft tumours was extracted, and the mRNA expression levels of *CYP1A1* and *CYP1B1* were assessed by RT‒qPCR (*n* = 6). *P < 0.05; ** *P* < 0.01; *** *P* < 0.001; **** *P* < 0.0001.

The induction of TDO2 upregulation by ADT in androgen-dependent prostate cancer raises the interesting question of whether TDO2 expression persists into the recurrent CRPC stage. We evaluated the TCGA and GTEx datasets and reported that *TDO2* mRNA levels were considerably higher in prostate cancer tissues than in normal control tissues (Fig. 3a) and increased with prostate cancer development and Gleason score (Fig. 3b). Interestingly, according to single-cell sequencing analysis of CRPC cell lines^37^, TDO2 was significantly overexpressed in a more advanced drug-resistant CRPC cell subpopulation (Fig. 3c; Supplementary Fig. S10a), suggesting that *TDO2* mRNA is persistently expressed in the late stage of prostate cancer. Immunohistochemical staining of prostate cancer tissues further revealed that TDO2 protein levels were substantially higher in CRPC and high-grade adenocarcinomas than in low-grade adenocarcinomas (Fig. 3d). A similar expression pattern was observed in prostate cancer cell lines, with TDO2 being more highly expressed in the advanced CRPC prostate cancer cell lines DU145 and PC3 (Fig. 3e; Supplementary Fig. S10b). Interestingly, AhR expression was also elevated in advanced prostate cancer cell lines (Fig. 3e; Supplementary Fig. S10c–e), whereas IDO1 was not detected in any of the cell lines that were studied (Fig. 3e). Therefore, TDO2 may be the primary tryptophan-consuming enzyme in prostate cancer. In addition, among the LNCaP xenograft tumours we collected, both TDO2 and AhR proteins were significantly upregulated in tumours that recurred after castration compared with normal tumours (Fig. 3f), and the mRNA levels of *TDO2*, *AhR*, and *CYP1A1* were also significantly elevated (Supplementary Fig. S10f). These results indicate that the TDO2-AhR pathway remained activated and persistently expressed in the stage of recurrent advanced prostate cancer following ADT.

To examine the role of persistent TDO2 expression in CRPC, we generated an enzalutamide-resistant LNCaP cell line by treating it with progressively higher concentrations of enzalutamide (MDV3100) over an extended period of time (Fig. 3g), which may recapitulate treatment-induced cellular dormancy and recurrence in vitro. Compared with those in parental LNCaP cells, the protein and mRNA expression levels of TDO2 and AhR were significantly elevated in the LNCaP-EnzR cell line (Fig. 3g; Supplementary Fig. S10b*−*e). We then knocked down TDO2 by shRNA in LNCaP-EnzR and PC3 cells and found that TDO2 knockdown significantly reduced cell proliferation, colony formation and cell migration (Fig. 3h–j; Supplementary Fig. S11a–e), suggesting that TDO2 promoted the aggressive progression of CPRC. As AhR was highly expressed in LNCaP-EnzR and PC3 cells, we used siRNA to knock down AhR in both cell lines and found that AhR knockdown reduced cell proliferation in a manner similar to TDO2 knockdown (Supplementary Fig. S11f, g). Pharmacologically targeting TDO2 or AhR with their inhibitors 680C91, CH223191 or BAY2416964 suppressed cell proliferation and colony formation in LNCaP-EnzR and PC3 cells (Fig. 3k; Supplementary Fig. S11h–j), and both 680C91 and CH223191 inhibited cell viability and colony formation in a wider range of CRPC cell lines (C4-2, 22RV1, and DU145) (Supplementary Fig. S12a–i), suggesting that TDO2-Kyn-AhR signalling promotes the aggressive growth of CRPC. Interestingly, the IDO1 inhibitor lindrodostat had no inhibitory effects on LNCaP-EnzR or PC3 cells (Supplementary Fig. S13a), which was consistent with the finding that prostate cancer cells expressed undetectable levels of IDO1 protein (Fig. 3e). Adding Kyn to the culture media of TDO2i-treated LNCaP-EnzR and PC3 cells restored both cell proliferation and migration (Fig. 3l; Supplementary Fig. S13b–d), indicating that Kyn was involved in mediating the aggressive behaviours (migration and proliferation) of recurrent CRPC cells that TDO2 promoted.

Additionally, the TDO2 inhibitor LM10 suppressed the growth of LNCaP-EnzR and PC3 xenograft tumours in vivo (Fig. 3m; Supplementary Fig. S14a–d). The AhR inhibitor CH223191 also strongly suppressed LNCaP-EnzR xenograft tumour growth (Fig. 3m; Supplementary Fig. S14a–c). However, the addition of Kyn diminished the inhibitory effect of LM10 on LNCaP-EnzR xenograft tumour growth in vivo but not that of CH223191 (Fig. 3m; Supplementary Fig. S14c), suggesting that Kyn participates in mediating the growth of TDO2-promoted recurrent CRPC in vivo. We then extracted protein and RNA from LNCaP-EnzR tumours and found that although the TDO2 inhibitor 680C91 and the AhR inhibitor CH223191 had no significant effect on AhR protein or mRNA levels (Supplementary Fig. S14e, f), they significantly reduced the mRNA levels of the AhR target gene *CYP1A1* (Fig. 3n). Furthermore, CH223191 also significantly reduced the expression of *CYP1B1*, another target gene of AhR (Fig. 3n), suggesting that AhR inhibition was more effective, which was consistent with the results of tumour growth suppression (Fig. 3m; Supplementary Fig. S14a–c). Taken together, these findings suggest that persistent TDO2 expression into the CRPC stage encouraged the aggressive progression of CRPC by controlling Kyn-AhR signalling.

### The switch from AR to GR mediates the sustained expression of TDO2 in CRPC

**Fig. 4:**
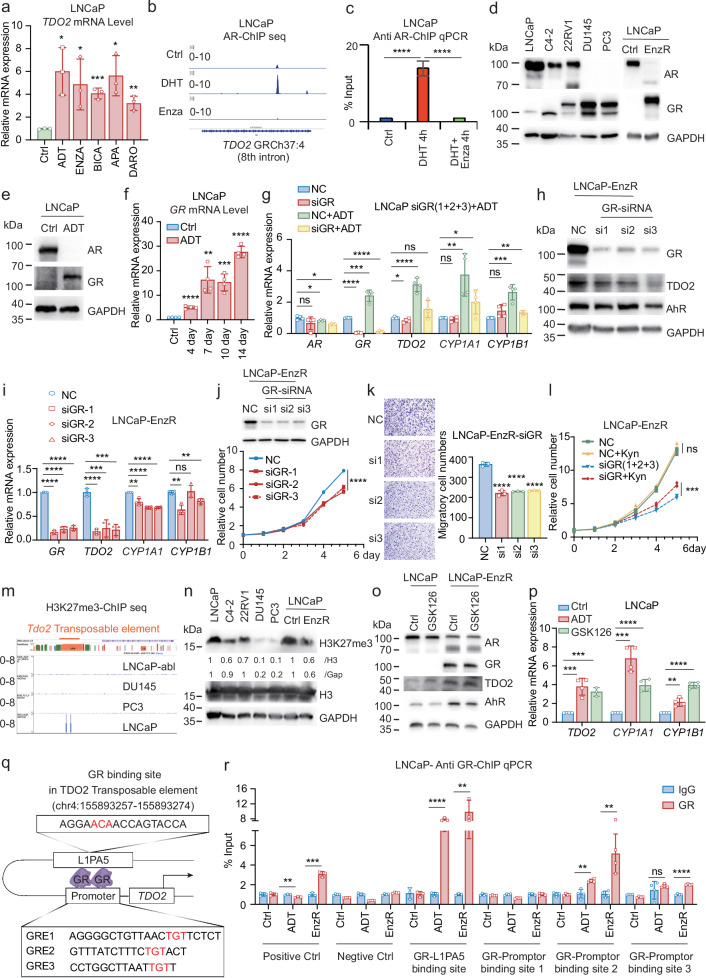
The AR-to-GR switch mediates the sustained expression of TDO2 in CRPC. **a** LNCaP cells were treated with ADT, enzalutamide, bicalutamide, apalutamide, or darolutamide, and the mRNA levels of *TDO2* were assessed by RT‒qPCR. **b** The binding of AR to the *TDO2* gene in LNCaP, LNCaP/DHT and LNCaP/enzalutamide cells was analysed by AR-ChIP sequencing data. **c** ChIP‒qPCR was used to verify the binding of AR to the TDO2 gene in LNCaP, LNCaP + DHT and LNCaP + DHT + enzalutamide cells. **d** The protein expression levels of AR and GR in the prostate cancer cell lines LNCaP, C4-2, 22RV1, DU145, PC3 and LNCaP-EnzR were examined by western blotting assay. **e** The protein expression levels of GR and AR in LNCaP cells subjected to ADT (2 weeks) were assessed by western blotting assay. **f** The mRNA levels of *Gr* were assessed by RT‒qPCR in LNCaP cells subjected to ADT for different durations (0, 4, 7, 10, and 14 days). **g** GR was silenced by siRNA in LNCaP cells with or without ADT for 48 h, and the mRNA levels of *AR, GR, TDO2, CYP1A1* and *CYP1B1* were examined by RT‒qPCR. **h** GR was knocked down by siRNA in the LNCaP-EnzR cell line, and the protein levels of GR, TDO2 and AhR were measured by western blotting assay. **i** The mRNA levels of *GR, TDO2, CYP1A1* and *CYP1B1* were measured RT‒qPCR after GR was knocked down by siRNA in LNCaP-EnzR cells. **j**, **k** GR was knocked down by siRNA in LNCaP-EnzR cells, the protein level of GR was measured by western blotting assay, and cell proliferation (**j**) and cell migration (**k**) were examined. **l** GR was knocked down with siRNA in LNCaP-EnzR cells, and cell proliferation was measured at 0, 1, 2, 3, 4, and 5 days in the presence or absence of Kyn. **m** H3K27me3 binding to the TDO2 genome in LNCaP, LNCaP-abl, DU145 and PC3 cell lines was analyzed by H3K27me3 ChIP sequencing data (data from http://cistrome.org/db). **n** The protein expression levels of H3K27me3 and H3 in the prostate cancer cell lines LNCaP, C4-2, 22RV1, DU145, PC3 and LNCaP-EnzR were measured by western blotting assay. **o** LNCaP and LNCaP-EnzR cells were treated with GSK126 for 48 h, and the protein expression levels of AR, GR, TDO2 and AhR were measured by western blotting assay. **p** LNCaP cells were treated with GSK126 alone or in combination with ADT for 48 h, and the mRNA levels of *TDO2, CYP1A1* and *CYP1B1* were measured by RT‒qPCR. **q** The TDO2 binding sequence regulated by GR was predicted on the regulatory element of TDO2 on the basis of the GR motif (binding site prediction website: https://jaspar.elixir.no/), which predicted three glucocorticoid response elements (GRE1–3) in the TDO2 promoter and one in the L1PA5 transposon. **r** The binding of GR to the *TDO2* regulatory element was verified by ChIP‒qPCR. *P < 0.05; ** *P* < 0.01; *** *P* < 0.001; **** *P* < 0.0001.

To investigate how ADT induces TDO2 upregulation in androgen-dependent prostate cancer, we first evaluated the mRNA levels of the TDO2-AhR axis after ADT. The mRNA levels of *TDO2*, *AhR*, and *CYP1A1* all increased significantly with ADT (Fig. 4a; Supplementary Fig. S15a–d), suggesting that ADT continuously activated the TDO2-Kyn-AhR pathway. However, the mRNA levels of *AR* and the AR pioneer factor *Forkhead box A1* (*FOXA1*) (but not *GATA2*) decreased significantly after ADT (Supplementary Fig. S15e–g). In addition, treatment with several anti-androgen agents (enzalutamide, bicalutamide, apalutamide and darolutamide) also significantly upregulated the mRNA levels of *TDO2*, *AhR*, and *CYP1A1* (Fig. 4a; Supplementary Fig. S15h, i). These results imply that AR may negatively regulate TDO2 transcription, thereby negatively modulating the TDO2-AhR pathway.

Bioinformatic analysis revealed that *TDO2* mRNA levels were negatively correlated with *AR* and genes associated with *AR*, including SAM pointed domain containing ETS transcription factor (*SPDEF*), *FOXA1*, cAMP responsive element binding protein 3 like 4 (*CREB3L4*), *RAB6C* (a member of the RAS oncogene family), NK3 homeobox 1 (*NKX3-1*) and coiled-coil domain containing 125 (*CCDC125*), in an RNA sequencing database comprised of 208 prostate cancer samples^38^ (Supplementary Fig. S16a, b). Chromatin Immunoprecipitation sequencing (ChIP-seq) analysis of AR revealed that, in the presence of DHT, AR bound to the eighth intron of *TDO2* in the LNCaP cell line^39,40^ (Fig. 4b; Supplementary Fig. S17a). We verified this analysis by a ChIP-quantitative real-time PCR (ChIP‒qPCR) assay (Fig. 4c), which suggests that androgen-activated AR binds to the intronic DNA of TDO2 and suppresses its transcription and that androgen deprivation releases this suppression and leads to TDO2 transcription. Furthermore, the observation that AR bound to the eighth intron of TDO2 in the presence of DHT was also observed in VCaP cells, a different androgen-dependent prostate cancer cell line (Supplementary Fig. S17b). Interestingly, in CRPC cells, AR no longer binds to the TDO2 recognition site (Supplementary Fig. S17c), suggesting that TDO2 at the CRPC stage may be controlled by a different transcription factor.

The establishment of an enzalutamide-resistant LNCaP cell line allowed us to investigate the underlying mechanisms by which TDO2 is persistently expressed in the recurrent CRPC stage. As shown in Fig. 3g, the expression of TDO2 and AhR was significantly elevated in the LNCaP-EnzR cell line, whereas that of the full-length AR was decreased to an undetectable level (Fig. 4d), corroborating the suggestion that another transcription factor might regulate persistent TDO2 expression in the CRPC stage. To identify the transcription factor, we examined the DNA sequences of the TDO2 regulatory element, including the promoter region, enhancers and transposons, with a transcription factor prediction programme (version 8.3 of TRANSFAC) and found that 25 transcription factors (factors predicted within a dissimilarity margin less than or equal to 1%) could bind to the TDO2 regulatory element and have the potential to transcribe TDO2 (Supplementary Fig. S17d). Among them, transcription factor 4 (TCF4) has been recently reported to regulate TDO2 transcription^22^. The GR was of interest to us, as it has been shown to be significantly expressed in CRPC and to mediate the castration resistance of prostate cancer^41^. We then examined GR expression in prostate cancer cell lines and found that as the disease progressed, GR expression gradually increased, whereas AR expression decreased (Fig. 4d; Supplementary Fig. S18a, b). Similarly, in LNCaP xenograft tumours (Fig. 2m; Supplementary Fig. S9b), compared with those in normal tumours, the protein and mRNA levels of AR decreased in tumours that recurred after castration treatment (Supplementary Fig. S18c, d), whereas the protein and mRNA levels of GR increased (Supplementary Fig. S18c, d). Furthermore, the expression of AR target genes was significantly reduced (Supplementary Fig. S18e), whereas the expression of GR target genes was significantly elevated (Supplementary Fig. S18e). Intriguingly, we observed that GR was markedly upregulated in LNCaP-EnzR cells (Fig. 4d). Like TDO2, ADT also caused the upregulation of GR mRNA and protein in LNCaP cells (Fig. 4e, f; Supplementary Fig. S18f), suggesting that GR is highly likely to take over the role of AR in regulating TDO2 in the dormant and recurrent CRPC stages.

To verify the regulatory effect of GR on the TDO2-AhR pathway, we first added the GR antagonist Cort108297 or the GR agonist dexamethasone to ADT-treated or untreated LNCaP and found that Cort108297 reversed the ADT-induced upregulation of *TDO2*, *AhR*, and *CYP1A1* mRNA (Supplementary Fig. S19a–c), whereas dexamethasone promoted the upregulation of *TDO2*, *AhR*, and *CYP1A1* mRNA under ADT conditions (Supplementary Fig. S19a–c). Moreover, similar to dexamethasone treatment, GR overexpression also significantly promoted the upregulation of *TDO2* and *CYP1A1* mRNA under ADT conditions (Supplementary Fig. S19d–g). Although GR overexpression had no significant effect on the cell cycle of LNCaP cells in response to ADT (Supplementary Fig. S19h), it increased the resistance of LNCaP cells to ADT (Supplementary Fig. S19i). Similarly, knocking down GR by siRNA led to the abrogation of ADT-induced upregulation of *TDO2*, *CYP1A1* and *CYP1B1* mRNA in LNCaP cells (Fig. 4g). In LNCaP-EnzR cells, dexamethasone also promoted the upregulation of *TDO2*, *AhR*, and *CYP1A1* mRNA (Supplementary Fig. S20a). However, although Cort108297 alone or in combination with the AhR inhibitor CH223191 significantly inhibited cell growth in LNCaP-EnzR cells (Supplementary Fig. S20b), Cort108297 was not as effective in blocking the TDO2-AhR pathway as it was in LNCaP cells (Supplementary Figs. S19a–c and S20a), suggesting that targeting GR was more effective in ADT-treated androgen-sensitive prostate cancer cells than in recurrent late-stage prostate cancer cells.

We then used siRNA to knock down GR in LNCaP-EnzR cells and detected significant decreases in TDO2 protein and mRNA levels (Fig. 4h, i). Interestingly, GR siRNA knockdown did not change the protein levels of AhR but lowered the levels of *CYP1A1* and *CYP1B1* (Fig. 4h, i), suggesting that GR regulated the expression of TDO2 to modulate the activity of AhR. GR siRNA knockdown decreased cell proliferation and migration (Fig. 4j, k; Supplementary Fig. S20c), which was rescued by the addition of Kyn to the culture media (Fig. 4l). Taken together, these findings suggest that AR suppressed TDO2 and GR transcription in androgen-sensitive prostate cancer cells, whereas ADT relieved AR suppression and resulted in TDO2 transcription and expression of GR, which then took over the role of AR to further activate TDO2 transcription and maintain the recurrent CRPC stage.

Epigenetic regulation has been shown to be involved in prostate cancer progression^42,43^. Two histone methylation H3K27me3 sites within the TDO2 regulatory region were detected in the hormone-sensitive prostate cancer cell line LNCaP but not in advanced CRPC cell lines (Fig. 4m). Both sites were located within the transposon element L1PA5 (Fig. 4m), with one on chromosome 4 (155893197–155893397) and the other on chromosome 4 (155894370–155894553) (Supplementary Fig. S21a). Interestingly, a GR-binding motif was also found in the transposon at chromosome 4 (155893257–155893274), which specifically overlapped with the first H3K27me3 site (Supplementary Fig. S21a, b), suggesting that GR may cooperate with H3K27me3 to regulate TDO2 transcription. It has been reported that GR can enlist histone demethylases to eliminate histone methylation and control the expression of genes^44^. Furthermore, the L1 transposon subfamily L1PA5 (Supplementary Fig. S21c) has substantial transcription factor binding activity for GR (NR3C1)^45^. We first examined H3K27me3 expression in LNCaP-EnzR cells and other prostate cancer cell lines and found that H3K27me3 was much lower in LNCaP-EnzR cells than in other late-stage prostate cancer cells (Fig. 4n; Supplementary Fig. S22a, b), suggesting that the decrease in H3K27me3 might be connected to the increased expression of TDO2 at the CRPC stage. We then treated LNCaP cells with the EZH2 inhibitor GSK126 to suppress H3K27me3 and observed considerable upregulation of TDO2 mRNA and protein (Fig. 4o; Supplementary Fig. S22c, d), whereas TDO2 expression did not change in LNCaP-EnzR cells treated with GSK126 (Fig. 4o). In addition, treatment of LNCaP cells with GSK126 also resulted in increased mRNA levels of the AhR downstream genes *CYP1A1* and *CYP1B1* (Fig. 4p), suggesting that EZH2 in LNCaP cells negatively regulated TDO2. Interestingly, three glucocorticoid response elements (GREs) have been reported in the promoter of TDO2 (Supplementary Fig. S23a)^46^, which suggests that the suppression of H3K27me3 in L1PA5 may promote the formation of a regulatory loop of GR with the TDO2 promoter and L1PA5 that activates TDO2 transcription (Fig. 4q). We confirmed that GR bound to the TDO2 transposable element and promoter element through the GR motif by ChIP‒qPCR (Fig. 4r; Supplementary Fig. S23b, c). Furthermore, ADT increased the binding of GR to the TDO2 transposable element and promoter binding site 2 (Fig. 4r), suggesting that GR takes over the role of AR in activating TDO2 transcription during the ADT course of treatment. This finding corroborated our observation that the siRNA-mediated knockdown of GR in LNCaP cells led to a decrease in ADT-induced TDO2 upregulation and AhR activation (Fig. 4g).

### Differential AhR signalling in ADT-induced dormant cells and recurrent cells

Although the same TDO2-Kyn-AhR pathway mediates both the protection of ADT-induced dormant cell death for androgen-dependent prostate cancer and the promotion of prostate cancer progression for recurrent CRPC, we assume that the setting for AhR activation is different and thus may activate the expression of different sets of genes under these two scenarios to allow for tumour dormancy and recurrence progression.

**Fig. 5:**
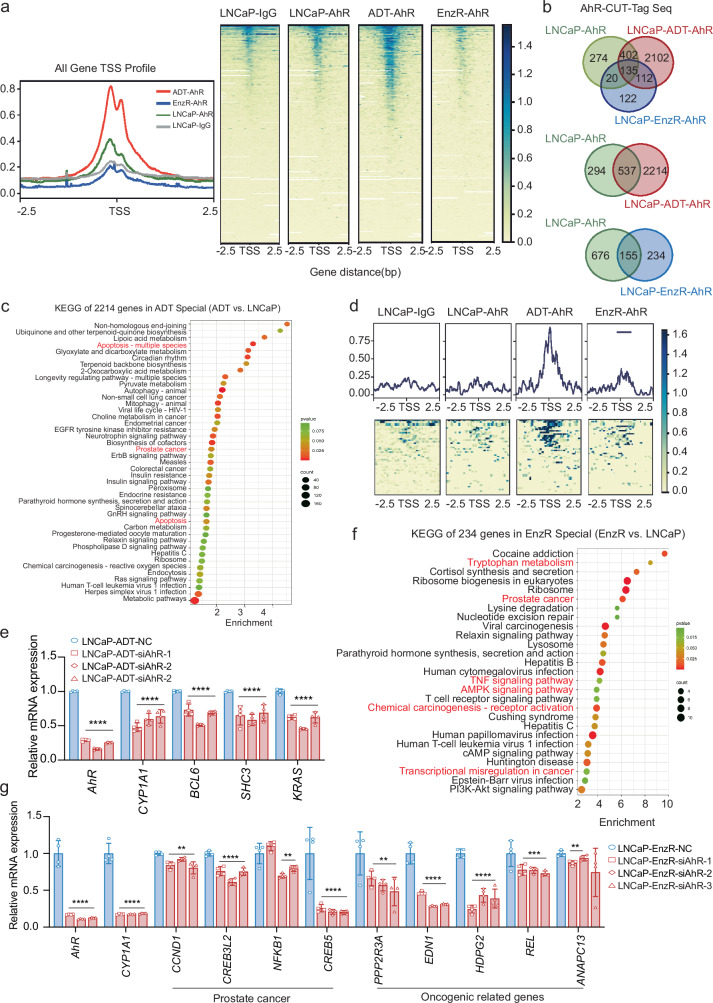
Differential AhR signalling in ADT-induced dormant cells and recurrent cells. **a** Transcription start state (TSS) average map and heatmap for CUT-Tag sequencing analysis. **b** CUT-Tag sequencing analysis was carried out on the transcription factor AhR in LNCaP cells, LNCaP+ADT cells and LNCaP-EnzR cells. After normalization, three sets of co-analyses and pairwise comparisons were performed to compare the intersection of genes with peaks greater than 100. **c** Kyoto Encyclopedia of Genes and Genomes (KEGG) enrichment analysis of 2214 differential AhR-regulated genes when the ADT-treated and LNCaP groups were compared. **d** Average map and heatmap of apoptosis-related gene sets. **e** The mRNA levels of *AhR, CYP1A1, BCL6, SHC3* and *KRAS* were measured after siRNA interference of AhR in LNCaP+ADT cells. **f** KEGG enrichment analysis of 234 differentially expressed AhR-regulated genes from comparison of the LNCaP-EnzR and LNCaP groups. **g** The mRNA levels of *CCND1, CREB3L2, NFKB1, CREB5, PPP2R3A, EDN1, HSPG2, REL*, and *ANAPC13* were measured after siRNA interference of AhR in LNCaP-EnzR cells. KEGG enrichment analysis tool: https://david.ncifcrf.gov/. *P < 0.05; ** *P* < 0.01; *** *P* < 0.001; **** *P* < 0.0001.

To identify the different sets of genes activated by AhR at different phases of tumour progression, we carried out CUT&Tag chromatin profiling to investigate the differential AhR signalling in three different cell conditions: LNCaP cells, LNCaP cells with 7 days of ADT and LNCaP-EnzR cells (Fig. 5a). After normalization, we found that 135 genes enriched primarily in spliceosome, ribosome biogenesis in eukaryotes, and ribosome biogenesis processes were regulated by AhR in all three cell conditions (Fig. 5a; Supplementary Fig. S24a), indicating the essential role of AhR signalling in prostate cancer. Among the 274 genes that AhR specifically regulates in LNCaP cells were cell cycle-related genes, including protein phosphatase 2 scaffold subunit Abeta (*PPP2R1B*), cyclin-dependent kinase inhibitor 2B (*CDKN2B*), origin recognition complex subunit 6 (*ORC6*), and cyclin-dependent kinase 6 (*CDK6*) (Supplementary Fig. S24b), all of which were associated with dormant cell development following treatment adaptation. A total of 247 genes overlapped between the LNCaP + ADT cells and the LNCaP-EnzR cells (Supplementary Fig. S24c) in which TDO2 was upregulated and AhR was activated, including the shared 135 genes that were enriched in basic RNA and protein biogenesis processes (Supplementary Fig. S24a) and another 112 genes that were enriched in metabolic pathways, the p53 signalling pathway and transcriptional misregulation in cancer (Supplementary Fig. S24d), suggesting that these shared common pathways may mediate similar functions of AhR signalling in ADT-induced dormancy maintenance and the progressive growth of recurrent CRPC cells.

We then used pairwise alignment of the CUT&Tag sequencing data to identify the different gene sets controlled by AhR (Fig. 5b). When the ADT-treated group was compared with the LNCaP group, 2214 distinct AhR-regulated genes were found. These genes were enriched primarily in 43 pathways, including apoptosis and prostate cancer pathways (Fig. 5c), suggesting that AhR might specifically activate the apoptosis pathway to respond to ADT. Indeed, apoptosis-related genes were enriched in the ADT group (Fig. 5c). BCL6 transcription repressor (*BCL6*), SHC adaptor protein 3 (*SHC3*) and *KRAS* (KRAS proto-oncogene, GTPase) in the antiapoptosis-related gene category have been shown to be more favored in the binding of AhR (Supplementary Fig. S25a). Among these genes, *BCL6* functions as a transcriptional repressor to epigenetically repress the expression of cell death genes and prevent apoptosis^47,48^. We verified by RT‒qPCR that *BCL6*, *SHC3* and *KRAS* were significantly upregulated in LNCaP cells after ADT (Supplementary Fig. S25b). Knocking down AhR by siRNA in LNCaP cells treated with ADT resulted in significant downregulation of the antiapoptotic genes *BCL6*, *SHC3* and *KRAS* (Fig. 5e), suggesting that one of the primary roles of the TDO2-Kyn-AhR pathway, which is activated in early prostate cancer following ADT, is to prevent apoptosis, enabling cancer cells to survive and maintain themselves as dormant cells.

When LNCaP-EnzR cells were compared with LNCaP cells, 234 distinct AhR-regulated genes were found in the enzalutamide-resistant cells (Fig. 5b), which were enriched in 27 pathways, including a number of oncogenic pathways, such as the TNF signalling pathway, the AMPK signalling pathway, chemical cancer receptor activation and transcriptional dysregulation in cancer (Fig. 5f). Tryptophan metabolism was one of the 27 pathways (Fig. 5f), and the downstream target genes of AhR, *CYP1A1* and *CYP1B1*, were considerably elevated after AhR binding (Supplementary Fig. S25c), suggesting that tryptophan metabolism is significantly activated in recurrent castration-resistant prostate cancer. Genes enriched in prostate cancer pathways, such as cyclin D1 (*CCND1*), cAMP responsive element binding protein 3 like 2 (*CREB3L2*), nuclear factor kappa B subunit 1 (*NFKB1*), cAMP responsive element binding protein 5 (*CREB5*), and oncogene-related genes, such as protein phosphatase 2 regulatory subunit B“alpha (*PPP2R3A*), endothelin 1 (*EDN1*), heparan sulfate proteoglycan 2 (*HSPG2*), *REL* (REL proto-oncogene, an NF-kB subunit), and anaphase promoting complex subunit 13 (*ANAPC13*), were among the representative genes chosen to specifically bind to AhR in enzalutamide-resistant recurrent cells (Supplementary Fig. S25d, e). siRNA-mediated knockdown of AhR in LNCaP-EnzR cells led to considerable downregulation of *CCND1*, *CREB3L2*, *NFKB1*, *CREB5*, *PPP2R3A*, *EDN1*, *HSPG2*, *REL*, and *ANAPC13* (Fig. 5g), strongly suggesting that AhR in advanced prostate cancer tumours is involved mainly in cancer cell growth and progression, in contrast to its function in early-stage androgen-dependent prostate cancer in response to ADT.

## Discussion

**Fig. 6:**
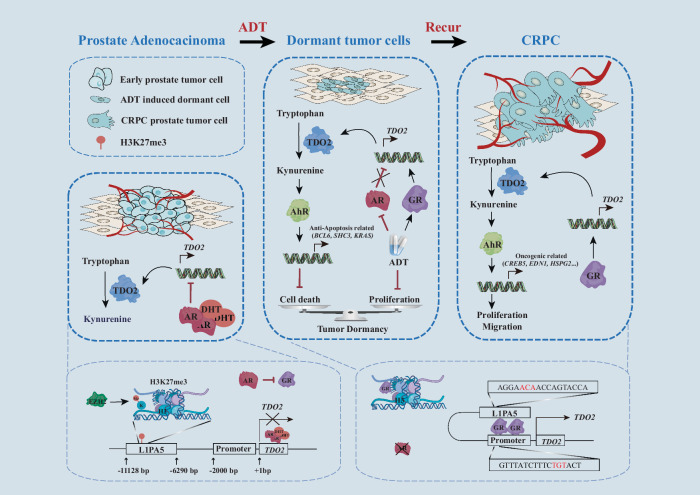
A working model. In androgen-dependent prostate adenocarcinoma, AR inhibits TDO2 transcription by binding to the TDO2 intron and suppresses GR expression. EZH2-mediated H3K27me3 on the TDO2 transposon (L1PA5) blocks GR binding and suppresses its transcriptional activity for TDO2 in androgen-sensitive prostate cancer. ADT results in dormant prostate cancer, in which AR expression is suppressed to alleviate the suppression of TDO2 transcription and concurrently increases GR expression to form a transcriptional loop between GR and GREs in the L1PA5 and TDO2 promoter, activating TDO2 transcription, which persists into the recurrent CRPC stage. TDO2 upregulation activates the Kyn-AhR pathway, which functions to maintain the survival of dormant tumour cells and encourages the onset of recurrence and the progression of castration-resistant prostate cancer.

In this study, we demonstrated that the tryptophan catabolism pathway TDO2-Kyn-AhR maintains the survival of ADT-induced dormant prostate cancer cells and promotes the recurrence and aggressive growth of CRPC cells (Fig. 6). Mechanistically, AR inhibits TDO2 transcription by binding to the TDO2 intron and suppresses GR expression; simultaneously, EZH2-mediated H3K27me3 on the TDO2 transposon (L1PA5) blocks GR binding and TDO2 transcription in androgen-sensitive prostate cancer. ADT reduces AR expression to alleviate the suppression of TDO2 transcription and concurrently increases GR expression to activate TDO2 transcription. The loss of H3K27me3 at the TDO2 transposon (L1PA5) allows GR to form a transcriptional loop between GREs in the L1PA5 promoter and the TDO2 promoter and subsequently activates TDO2 transcription and the Kyn-AhR pathway, which functions to maintain the survival of dormant tumour cells and to encourage the onset of recurrence and the progression of prostate cancer to the CRPC stage (Fig. 6). Therefore, our findings reveal a critical role for TDO2-Kyn-AhR signalling in ADT-induced tumour dormancy and recurrence in prostate cancer and suggest a new therapeutic target for androgen-sensitive prostate cancer by combining ADT with targeting the TDO2-Kyn-AhR pathway.

A notable characteristic of tumour dormancy, especially cellular dormancy, is quiescence, i.e., dormant tumour cells remain in the G0‒G1 phase of the cell cycle. Our results revealed that short-term treatment with ADT (7–21 days) in LNCaP cells resulted in a quiescent phenotype (G0‒G1 phase) (Fig. 1a), suggesting that a temporary dormant state was induced by ADT, as the addition of androgen restored the growth of the cells. The standard dormancy marker P27 was upregulated by ADT, strongly suggesting that ADT is able to induce dormant cancer cells. Most recently, Dong et al. used ADT-induced PDX dormancy models to demonstrate that the development of recurrent tumours may require the development of adaptive mechanisms to exit dormancy and regrow in an unfavourable androgen-suppressed environment^10^. In this study, we showed that TDO2 was upregulated after short-term ADT and was persistently expressed in the recurrent CRPC stage, which may suggest an adaptive mechanism to allow dormant cells to exit dormancy and regrow, i.e., TDO2 upregulation activates AhR through Kyn to protect dormant cells from ADT-induced cell death, and the AR-to-GR switch persistently increases TDO2 expression to exit dormancy and promote the recurrence and aggressive growth of dormant cells via the same TDO2-Kyn-AhR pathway.

In *Pten*^−/−^ cKO mice, the expression of TDO2 was significantly upregulated and further elevated in castrated *Pten*^−/−^ cKO mice (Fig. 1j, k). The loss of PTEN in prostate epithelial cells has been reported to inhibit the expression of androgen-responsive genes and AR transcriptional outputs, as PTEN can regulate AR transcription factor activity by modulating the expression of EGR1, c-JUN, and EZH2^35^. These findings suggest a potential mechanism by which TDO2 is highly expressed in *Pten*^−/−^ cKO mice. We demonstrated that TDO2 expression is negatively regulated by AR (Fig. 1g), and the loss of PTEN resulted in the upregulation of TDO2 expression through the negative regulation of AR transcriptional activity^37^, which therefore relieved the suppression of TDO2 by AR. In recurrent tumours, GR replaces AR to activate a set of similar yet distinguishable target genes, which is crucial for maintaining the resistant phenotype^41^, and this is also an important reason for the sustained high expression of TDO2 in recurrent tumours.

The establishment of the LNCaP-EnzR cell line in this study through treatment with a series of concentrations of enzalutamide allowed us to establish an in vitro model of ADT-induced cellular dormancy and recurrence. Our findings that GR and TDO2 were significantly upregulated in enzalutamide-resistant cells suggest that treatment-induced dormant LNCaP cells may utilise the TDO2-Kyn-AhR signalling axis as an adaptive mechanism to maintain dormancy and reactivation, whereas the switch from the transcription factor AR to GR is the driving force involved in this process.

GR overexpression has been shown to be an AR-independent mechanism that drives resistance to anti-androgen therapy^41^. Mechanistic studies of GR overexpression indicate that GR expression per se is subject to negative AR regulation^49^, as AR binds to an intronic enhancer of GR and directly represses GR expression in a subpopulation of prostate cancer cells^50^, similar to TDO2 regulation in this study. We have shown that in LNCaP cells, ADT induces the mRNA and protein expression of GR (Fig. 4e, f), which is consistent with a previous report that when AR is suppressed by ADT or enzalutamide, the inhibition of GR expression is reversed, and GR expression is amplified^50^. AR and GR can recognise a similar regulatory element corresponding to the canonical AR binding motif or GR-binding motif (Supplementary Fig. S23b), which enables GR to bind to AREs and modulate some of the classical androgen target genes^51^. Moreover, AR and GR share a significant number of chromatin binding sites^52,53^. We detected an ARE in the 8^th^ intron of TDO2 and four GREs in the TDO2 regulatory elements (Fig. 4b; Supplementary Fig. S17a and S21a and S23a), suggesting TDO2 regulation by both AR and GR. We demonstrated that AR binds to the TDO2 intron to suppress its transcription and that ADT relieves this suppression, whereas GR binds to TDO2 GREs (one located in a TDO2 transposon and three in the TDO2 promoter) to form a regulatory loop and promote TDO2 transcription (Fig. 4q). We further demonstrated that the decrease in H3K27me3 in L1PA5 promotes the formation of a regulatory loop of GR, the TDO2 promoter and L1PA5 to activate TDO2 transcription (Fig. 6). Together, these findings suggest a molecular mechanism to regulate TDO2 upregulation by ADT and allow it to persist into the recurrent CRPC stage: AR suppresses TDO2 transcription, and ADT relieves this suppression, while concurrently, ADT also relieves GR suppression by AR and upregulates GR expression to activate TDO2 transcription, which persists until the recurrent CRPC stage. Therefore, our study reveals a novel mechanism to regulate TDO2 and characterises a new cellular function of the TDO2-Kyn-AhR signalling axis that is required for maintaining ADT-induced cellular dormancy and promoting dormancy exit-to-reactivation and thus recurrence.

Although we have demonstrated that prostate cancer cells use the same TDO2-Kyn-AhR signalling pathway to both protect dormant cells from ADT-induced cell death and promote the aggressive growth of recurrent prostate cancer cells in different phases of tumour progression, this pathway clearly activates different sets of genes to modulate tumour dormancy and recurrence. By using the CUT&Tag profiling technique, we identified different sets of genes under these two scenarios: ADT-induced TDO2-Kyn-AhR signalling activated more apoptotic protective genes that promoted the survival of dormant cells and maintained their dormant state, whereas TDO2-Kyn-AhR signalling in recurrent CRPC activated more proliferative oncogenes to facilitate dormancy exit and aggressive growth of recurrent cancer cells (Fig. 5). The different functions of the same TDO2-Kyn-AhR signalling pathway in tumours may be caused by two distinct mechanisms: (1) epigenetic regulation is one potential mechanism, in which the epigenetic modifications in early-stage prostate cancer differ from those in late-stage recurrent prostate cancer; for example, the levels of H3K27me3 varied in LNCaP and LNCaP-EnzR cells treated with ADT, leading to AhR transcribing different sets of genes, which were modified by different epigenetic marks; (2) in the two different stages of prostate cancer, AhR may cooperate with different cofactors to activate different gene sets; for example, LNCaP and LNCaP-EnzR cells treated with ADT expressed different levels of the transcription factors AR and GR, which may collaborate with AhR to activate different genes, whereas in breast cancer, AhR can reactivate LINE-1 retrotransposons silenced by DNA methylation by regulating the TGF-β signalling pathway, promoting tumorigenesis and disease progression^54^.

Finally, our study identified TDO2 as a possible therapeutic target for early-stage prostate cancer when combined with ADT. Although TDO2 is highly expressed in the liver, which may hinder the development of TDO2 small molecule inhibitors, our study may advocate targeting TDO2-Kyn-AhR signalling as a new adjuvant therapy for androgen-sensitive prostate cancer patients treated with ADT.

## Materials and methods

### Cell culture, cell proliferation assay, cell death assay, cell cycle assay, cell apoptosis assay, colony formation assay, cell scratch assay, Transwell assays, gene silencing, and gene overexpression

The cells were maintained under standard or specific conditions (for details, see the Supplementary Information Text). The cells used in each experiment were subjected to identical and well-controlled conditions to test a specific hypothesis. However, conditions such as the starting cell numbers and the sizes of the tissue culture plates were not identical among the different experiments described in the different figures. Therefore, the results of different experiments may not be directly comparable. shRNA lentiviral plasmids and CRISPR single guide RNA (sgRNA) plasmids were either purchased from commercial sources or kindly provided by our collaborators. The detailed protocols are described in Supplementary Information.

### FUCCI

Two plasmids, Orange-hCdt1 and Green-hGem, were used^55^. We transfected these two plasmids into LNCaP cells and allowed fluorescent protein expression. The cells were subsequently treated with FBS or ADT, and the proportion of cells expressing red fluorescence or green fluorescence was subsequently determined via fluorescence microscopy.

### Quantitative real‑time PCR (qPCR)

Total RNA was isolated with the FastPure Cell/Tissue Total RNA Isolation Kit V2 (Vazyme, China) and then reverse transcribed into cDNA with TransScript II One-Step gDNA Removal and cDNA Synthesis SuperMix (TransGen, China). RT‒PCR was performed with AceQ qPCR SYBR Green Master Mix (Vazyme, China) on a QuantStudio 7 Flex Real-Time PCR system (Applied Biosystems). GAPDH served as an internal control in all the experiments. The sequences of the primers used for qPCR are listed in Supplementary Information.

### ChIP‒qPCR assay

The cells were crosslinked with 1% formaldehyde for 10 min at room temperature (RT), and the reaction was stopped by the addition of 125 mM glycine at RT for 5 min. The cells were subsequently washed with cold PBS and resuspended in cell lysis buffer (1% SDS, 1 mM EDTA, and 25 mM Tris–HCl, pH 8.0) for 30 min at 4 °C. The samples were subsequently centrifuged to obtain the supernatant. The supernatant was subjected to sonication to shear the chromatin to lengths between 100 and 500 bp. After centrifugation at 12,000 rpm for 15 min at 4 °C, the protein‒DNA complexes were immunoprecipitated with 2 μg of IgG or specific antibodies overnight at 4 °C, followed by further incubation with protein A&G magnetic beads for 2 h at 4 °C. The complexes were washed with low-salt, high-salt and LiCl wash buffers sequentially, followed by two washes with Tris-EDTA buffer at 4 °C. The Tris-EDTA complex was eluted by adding 100 μL of elution buffer (1% SDS, 0.1 M NaHCO_3_) with rotation at 37 °C for 30 min twice. Then, reverse crosslinking was carried out by adding NaCl (0.2 M) and proteinase K (0.5 mg/mL), and the mixture was incubated at 65 °C overnight. DNA was purified with a DNA purification kit. The purified DNA was dissolved in ddH2O for qPCR. The sequences of the primers used for ChIP‒qPCR are listed in Supplementary Information.

### Cell nucleus and cytoplasm separation

The extraction of the nucleus and cytoplasm was carried out according to the experimental steps outlined in the procedures of the NE-PER™ Nuclear and Cytoplasmic Extraction Reagent (#78833, Thermo Fisher Scientific, USA). The extracted samples were used for subsequent immunoblotting experiments.

### Western blotting assay

Total protein lysate was collected by lysing adherent cells with RIPA buffer (G-clone, China) supplemented with phosphatase and protease inhibitor cocktail (G-clone, China). The lysates were cleared by centrifugation, and the protein concentration in the supernatant was determined by a Bradford assay kit (Sangon Biotech, China). Equal amounts of total protein were used for immunoblotting. After electrophoresis, the proteins were transferred to polyvinylidene difluoride (PVDF) membranes, which were subsequently blocked in 5% nonfat milk. The samples were subsequently incubated with the following primary antibodies: anti-TDO2 (1:300, Proteintech, USA), anti-IDO1 (1:1000, Cell Signaling Technology, USA), anti-AR (1:1000, Cell Signaling Technology, USA), anti-GR (1:1000, Cell Signaling Technology, USA), anti-AhR (1:1000, Cell Signaling Technology, USA), anti-PSA (1:1000, Cell Signaling Technology, USA), anti-P27 (1:1000, Cell Signaling Technology, USA), anti-laminB1 (1:1000, Proteintech, USA), anti-β-tubulin (1:1000, A12289, ABclonal, China), anti-β-actin (1:3000, Cell Signaling Technology, USA) and anti-GAPDH (1:3000, Cell Signaling Technology, USA). After the membranes were washed with TBST (TBS with 0.1% Tween), the corresponding secondary antibodies conjugated with horseradish peroxidase (HRP) were incubated with the membranes. A Western Bright ECL kit (Advansta, USA) was used for protein detection.

### IHC staining

IHC staining was performed as described in a previous study^31,56^. In brief, paraffin sections of 5 μm thickness were prepared from tissue microarrays or xenograft tumours. The prostate tissue microarrays used in this study have been described previously^57,58^. The sections were sequentially deparaffinized and rehydrated with xylene through a graded ethanol series. Endogenous peroxidase activity was blocked in 3% H_2_O_2_ for 10 min, and the samples were boiled in 10 mM citrate buffer (pH 6.0) for 10 min for heat-induced antigen retrieval. A rabbit polyclonal anti-TDO2 antibody (1:200, Proteintech) was used as the primary antibody. IHC staining was quantified via Q score following the protocol described in a previous study^31,56^.

### Immunofluorescence staining of cells

The cells were spread onto a cell culture dish (Biosharp), fixed in methanol for 15 min, permeabilised with 0.1% Triton X-100, and then blocked with 1% BSA for 30 min. The cells were then incubated with a primary antibody against AhR (1:200, 28727-1-AP, Proteintech, USA) at 4 °C overnight. After being washed three times, the cells were probed with MultirAb™ CoraLite^®^ Plus 488-Goat Anti-Rabbit (1:1000, RGAR002, Proteintech, USA) for 1 h at RT, followed by nuclear counterstaining with DAPI (Beyotime, Shanghai, China). A sealing agent (Thermo Fisher Scientific, USA) was used for sealing. Images were obtained under a laser confocal microscope.

### Mouse xenograft models

Male NSG mice (4–6 weeks old) were purchased from Vital River (Charles River China, Beijing, China). For the castration recurrence experiments, LNCaP cells were prepared at a 1:1 ratio with Matrigel, and each male NSG mouse was subcutaneously injected with 200 µL of a mixture of 5 × 10^6^ LNCaP cells and Matrigel. Three weeks after tumour injection, the mice were randomly divided into a castrated group and a non-castrated group and were given a placebo, LM10 (20 mg/kg, orally) or CH223191 (15 mg/kg, orally). The animals were sacrificed after significant tumour recurrence or when the tumour volume exceeded 1000 mm^3^. Tumour growth was measured with callipers every three days, and the volume of each tumour was normalised to the volume on Day 0 (castration day). For drug experiments, LNCaP-EnzR cells were prepared in a 1:1 ratio with Matrigel, and each male NSG mouse was subcutaneously injected with 200 µl of a mixture of 1 × 10^6^ cells and Matrigel. When the tumour size reached 100 mm^3^, the mice were randomly assigned to receive a placebo, LM10 (20 mg/kg orally) or CH223191 (15 mg/kg orally). The tumour volume (mm^3^) was calculated as (length × width^2^)/2, and the animals were sacrificed when the tumour volume exceeded 1000 mm^3^. Tumour growth was measured with callipers every two days. Matrigel (354248) was purchased from Corning (Corning, New York, USA).

### Tumour protein extraction

The tumours were removed from liquid nitrogen and ground into powder in a grinder filled with liquid nitrogen. Cell lysates (Beyotime, China) containing protease inhibitors (MCE, New Jersey, USA) and phosphatase inhibitors (MCE, New Jersey, USA) were added, and sonication-assisted adequate lysis was performed to release proteins. The homogenised liquid is decoupled at 4 °C at high speed (10,000–14,000 *g*, 15–20 min). The centrifuged supernatant (containing protein) was transferred to a new centrifuge tube. The protein concentration was determined with a BCA protein concentration assay kit (Thermo Fisher Scientific, USA). The proteins were stored in a −80 °C freezer or subjected to subsequent experiments.

### Tumour RNA extraction

The tumours were removed from liquid nitrogen and ground into powder in a grinder filled with liquid nitrogen. Total RNA was isolated with the FastPure Cell/Tissue Total RNA Isolation Kit V2 (Vazyme, China).

### Motif prediction

The motifs of GR and AR were predicted by JASPAR (https://jaspar.genereg.net/) and aligned to the sequences in the TDO2 genome to identify specific regulatory gene sites.

### Bioinformatics analysis

To assess the clinical significance of TDO2 in prostate cancer, prostate cancer RNA-seq data and clinical data were downloaded from the GTEx portal database and TCGA.

### Metabolite extraction and mass spectrometry

As described previously^31^, 1 × 10^5^ cells were plated in 6-well plates. After the cells had settled, for experiments with LNCaP cells subjected to ADT, 2 ml of RPMI 1640 medium (Gibco) with either regular FBS or CS-FBS was used to replace the original culture medium after the cells were washed with cold PBS. The corresponding medium was changed every five days. At each time point, for intracellular metabolite measurement, the plates were placed on dry ice immediately. The medium was aspirated, and 80% HPLC grade methanol/water was added. For measurement of metabolite extraction from the medium, the medium was collected in an Eppendorf tube. Prechilled methanol (100%) was added to achieve a final methanol concentration of 80%. The plates or tubes were subsequently transferred to a −80 °C freezer for 15 min, after which the cells were scraped into extraction solvent. After being centrifuged for 10 min, the supernatant was transferred to a new Eppendorf tube. A speed vacuum was used to dry the samples at RT. The dry pellets were subsequently sent for further LC‒MS analysis. The data were normalised to the number of cells.

### CUT&Tag-seq assay

The CUT&Tag assay was performed with a Hieff NGS^®^ G-Type In-Situ DNA Binding Profiling Library Prep Kit for Illumina (#12598, Yeasen Biotechnology, Shanghai, China) according to the manufacturer’s instructions. Briefly, LNCaP cells and LNCaP cells treated with ADT for 7 days and LNCaP-EnzR cells were collected. Activated paramagnetic concanavalin A beads were added to the cells, followed by incubation with primary or secondary antibodies at RT. The cells were washed twice with a magnet stand to remove unbound antibodies and incubated with 1 μL of pA/G-transposome mixture for 1 h at RT. Next, the cells were resuspended in tagmentation buffer and incubated at 37 °C for 1 h. Then, 2 μL of 15× Terminate Solution, 1 μL of DNA Spike-in mix and 1 μL of 30× proteinase K were added to 31 μL of sample, followed by incubation at 55 °C for 30 min. To amplify the libraries, 20 µL of DNA was mixed with 3 µL of PCR Primer Mix, 1 µL of N502, 1 µL of N701 and 25 µL of 2× Ultima amplification mixture. The samples were subsequently placed in a thermocycler with a heated lid and subjected to the following cycling conditions: 72 °C for 3 min; 95 °C for 30 s; 15 cycles of 95 °C for 10 s, 55 °C for 30 s and 72 °C for 30 s; and a final extension at 72 °C for 5 min, followed by holding at 4 °C. Post-PCR clean-up was performed by adding a 1.2× volume of DNA selection beads, and the libraries were incubated with the beads at RT for 5 min, which were washed twice gently in 80% ethanol, followed by elution in 21 µL ddH_2_O. Paired-end Illumina sequencing was performed at the Shenzhen Gene+ medical laboratory.

### Statistical analysis

All experiments were performed three or more times, and the data are presented as the means ± standard deviations (s.d.). Statistically significant differences between two groups were assessed with two-tailed paired or unpaired *t*-tests. *P* < 0.05 was considered significant. For gene expression correlation, Pearson's r correlation analysis was used. All the statistical analyses were performed with GraphPad Prism software and Microsoft Excel. Figures were organized with Adobe Illustrator 2021.

## Supplementary information

Supplementary Information

## Data Availability

All the data generated or analysed during this study are included in the article and/or Supplementary Data.
