# Supplementary material for: AR to GR switch modulates differential TDO2-Kyn-AhR signalling to promote the survival and recurrence of treatment-induced dormant cells in prostate cancer
Source: Cell Discov. 2025 Aug 5;11:67. doi: 10.1038/s41421-025-00817-w (PMC12322048; doi:10.1038/s41421-025-00817-w)
Supplement: Supplementary file 1 — Supplementary Information [file 41421_2025_817_MOESM1_ESM.pdf]

AR to GR Switch Modulates Differential TDO2-Kyn-AhR Signaling to Promote the Survival and Recurrence of Treatment-induced Dormant Cells in Prostate Cancer

Sangsang Li[1, 7], Yifan Zhang[1,2, 7], Maoxing Luo[1], Weiwei Zhou[1], Yitong Chen[1], Dinglan Wu[3], Qiang Wei[2], Yan Chang[4,5, *] and Hailiang Hu[1,6,,8*]

1. Extended Materials and Methods
2. Supplementary Fig. S1-S25
3. Supplementary Figure legends
4. Supplementary Table S1

Extended Materials and Methods

Cell Culture

LNCaP, C4-2, 22RV1, DU145 and PC3 cells were purchased from Procell Life Science and Technology Co., Ltd. 293T was purchased from the Cell Bank of the Chinese Academy of Science (Shanghai, China). LNCaP-EnzR was a gift from the Department of Urology, Southern Medical University, China. All the cell lines are routinely checked for morphological and growth characteristics.

LNCaP, C4-2, 22RV1, DU145, PC3 and LNCaP-EnzR cells were cultured in RPMI-1640 medium (Gibco, USA) supplemented with 10% fetal bovine serum (FBS, Biological Industries, Israel) and 1% 100 unit /ml penicillin/streptomycin (Gibco, USA). Androgen deprived therapy (ADT) in LNCaP and was carried out by using RPMI 1640 (Gibco phenol red free), supplemented with 10% charcoal dextran stripped fetal bovine serum (CSS; Biological Industries, Israel) and 1% 100 unit /ml penicillin/streptomycin (Gibco, USA). 293T cells were cultured in DMEM medium (Gibco, USA) supplemented with 10% fetal bovine serum and 1% 100 unit /ml penicillin/streptomycin (Gibco, USA). All the cells were maintained at 37 °C with 5% CO2.

Cell proliferation assay

Cells were plated in 6-well plates. For LNCaP cells, $4*10^4$ cells were seeded in 2 mL medium while for C4-2, PC-3 and LNCaP-EnzR cells, $1*10^4$ cells were seeded initially. Cells were treated according to the experimental conditions (Treatment concentration of L-Kynurenine 100 μM, 680C91 10 μM, CH223191 10 μM and GSK126 10 μM). The fresh medium was replaced every 3 days. The cells were counted by flow cytometry (NovoCyte Agilent. USA). Prepare duplicate samples and average the count.

CCK8 assay was performed in 96-well plates with initial cell number of 2000/well. The cells were treated according to the experimental conditions (for PC3 and C4-2 cell lines: the therapeutic concentration gradient of 680C91 was 0 μM,0.1 μM,0.5 μM,2 μM,10

μM and 20 μM, the therapeutic concentration gradient of 1-MT was 0 μM, 600 μM, 1200 μM, the therapeutic concentration gradient of CH223191 was 0 μM,0.1 μM,0.5 μM,2 μM,10 μM and 20 μM; For LNCaP-EnzR cell line: 680C91 treatment concentration gradient was 0 μM, 1 μM, 2 μM, 5 μM, 10 μM and 20 μM; CH223191 treatment concentration gradient was 0 μM, 1 μM, 2 μM, 5 μM, 10 μM and 20 μM; 10 μl of Cell Counting Kit-8 reagent (Beyotime, China) was added to each test well, and OD450 of each well was measured on a microplate reader (BioTek, SH1M, USA). Prepare duplicate samples and average the count.

Cell death assay

Cells that were plated into six-well plates one day in advance were incubated with Incucyte® Cytotox green dye in the Incucyte® Live Cell Assay System in an incubator. Incucyte® Cytotox Green dye can label dying cells green, and Cytotox dye will enter the cell and fluorescently label the nucleus. The appearance of green-labeled nuclei over time allowed identification and quantification of dying cells. The proportion of dead cells was quantitatively analyzed by label-free counting of viable cells.

Cell cycle assay

The cytotoxicity of LNCaP cells or C4-2 cells to agents was determined by the Cell cycle and apoptosis detection kit (Beyotime, China). Briefly, drug-treated tumor cells were resuspended and fixed in 70% ethanol at 4ºC for 30 min or longer. The cells were stained with PI staining solution for 30 min at 37 ° C. Then cells cycle was analyzed on a NovoCyte (Agilent., USA). Each experiment was repeated for three independent times.

Cell apoptosis assay

The cytotoxicity of LNCaP cells to agents was determined by the Annexin V-FITC/ PI apoptosis detection kit (Beyotime, China). Briefly, agents treated tumor cells were resuspended and stained with Annexin V/PI staining solution for 20 min at room

temperature. Then cells apoptosis was analyzed on a NovoCyte (Agilent., USA). Each experiment was repeated for three independent times.

Cell lipid peroxidation assay

The lipid peroxidation detection kit (BODIPY 581/591 C11) (Beyotime, China) was used to assess the effect of the drug on lipid peroxidation in LNCaP cells. In brief, the drug-treated tumor cells were resuspended and incubated with BODIPY staining solution at 37 degrees Celsius for 20 minutes. Subsequently, cellular lipid peroxidation was detected using NovoCyte (Agilent, USA). Each experiment was independently repeated three times.

Colony formation assay

1×10^4 Cells were seeded into 6-cm dishes and grown for up to 10-14 days for colony formation. Cell culture media was refreshed every 3 days and colonies were fixed with cold 4% paraformaldehyde and then stained with 1% crystal violet. The number of colonies was imaged with camara and counted with ImageJ.

Cell scratch assay

The cells were plated one day in advance and the fusion rate reached 80% after 24 hours. Plates were scratched vertically with a six-well plate using a 200-microliter gun tip, and the scratched cells were washed with PBS. The drug to be tested was added, and samples were taken at 0, 24, and 48h time points and photographed. Image J software was used to analyze the mean value of scratch area.

Transwell assays

The cells starved with serum free media overnight were seeded at a density of 1×10^5 cells per well into invasion chambers (8-μm pore size, BD Biosciences). The lower chambers contained culture media containing 10% FBS. After incubation for 24 h, the wells were washed with PBS and fixed with 4% paraformaldehyde. The cells on the apical side of each insert were removed by scraping. Cells migrated to the basal side of the membrane were stained with 0.1% crystal violet. Randomly selected fields were

photographed and stained cells were counted, and student t-test was used for statistical analysis.

Lentiviral production and infection

A lentiviral vector bearing the shRNA or cDNA of interest, along with a packing vector (psPAX2, Addgene cat. #12260) and an envelope vector (pMD2.G, Addgene cat. #12259) was co-transfected into 293T cells using polyethyleneimine. Supernatants containing virus particles were collected at 48 h post transfection and filtered through 0.45 μm filters to remove cell debris. The viruses were used to infect target cells. Infected cells were then obtained in the presence of 2 μg/mL puromycin.

CRISPR knockout assays

The lentiCRISPRv2 knockout plasmid (Addgene cat. #52961) of TDO2 was constructed according to the Target Guide Sequence Cloning Protocol of Zhang Feng's laboratory. The Oligos are as follows:

TDO2-sgRNA#1-Forward: 5’ CACCGTCGCAGGTAGTGATAGCCTG 3’

TDO2-sgRNA#1-Reverse: 5’ AAACCAGGCTATCACTACCTGCGAC 3’

Lentiviral plasmids were packaged into lentivirus and infected into cells. 24 hours post-infection, cells were selected with 2 μg/ml puromycin for 7 days. Puromycin-resistant cells were pooled, amplified and analysis. Protein knockdowns were confirmed by western blotting.

shRNA knockdown assays

TDO2 knockdown was achieved by stable transduction with shRNA lentivirus. The cloning vector pLKO.1 is a gift from Hai Rao Lab, School of Medicine, Southern University of Science and Technology. The plasmid of TDO2 shRNA was constructed according to the protocol of pLKO.1 on Addgene.

(https://www.addgene.org/protocols/plko/).

All DNA sequences were verified by sequencing. The shRNA Oligos are as follows:

TDO2-shRNA#1-Forward:

5'CCGGAATCTGATTCATCACTGCTCTCGAGAGCAGTGATGAATCAGATTT TTTTG 3'

TDO2-shRNA#1-Reverse:

5'AATTCAAAAAAATCTGATTCATCACTGCTCTCGAGAGCAGTGATGAATC AGATT 3'

TDO2-shRNA#2-Forward:

5'CCGGAAATCTACAAATACCTTGTCTCGAGACAAGGTATTTGTAGATTTTT TTTG 3'

TDO2-shRNA#2-Reverse:

5'AATTCAAAAAAAATCTACAAATACCTTGTCTCGAGACAAGGTATTTGTA GATTT 3'

Lentiviral plasmids expressing shRNAs were packaged into lentivirus and infected into cells. 24 hours post-infection, cells were selected with 2 μg/ml puromycin for 7 days. Puromycin-resistant cells were pooled, amplified and analysis. Protein knockdowns were confirmed by western blotting.

siRNA knockdown experiments

siRNA for GR, TDO2 and AhR were purchased from Sangon Biotech (China). Cells were changed to fresh medium 4-6 h after transfection of siRNA, Protein knockdown were confirmed by western blotting. RT-qPCR was started 48 h after transfection, and WB was started 72h after transfection.

Overexpression of genes by lentivirus

For TDO2 and GR overexpression, pCDH-GFP vector was purchased from Miaolingbio (China). The cDNA of TDO2 and GR was amplified from the cDNA of PC3 and constructed into the plasmid pCDH-GFP vector. pCDH-GFP and pCDH-GFP-

TDO2/ pCDH-GFP-GR plasmid packaged into lentivirus for cell infection. proteins overexpression was confirmed by western blot or RT-qPCR.

Reagents

L-Tryptophan（T8941-25G）was purchased from Sigma-Aldrich (Germany). L-Kynurenine (HY-104026), 680C91 (HY-108681), CH-223191 (HY-12684), BAY2416964 (HY-135829), Bicalutamide (HY-14249), Apalutamide (HY-16060), Darolutamide (HY-16985), Linrodostat (HY-101560), Cort108297 (HY-15710) and Dexamethasone (Hexadecadrol) (HY-14648) were purchased from MedChemExpress (MCE, New Jersey, USA). LM10 (T4410) was purchased from TargetMol (Shanghai, China). GSK126 (S7061) was purchased from Selleck (Houston, Texas, USA).

Quantitative real-time PCR (qPCR)

The primer sequences used were as follows:

AR-Forward: 5’-GCTCCGCTGACCTTAAAGACATCC-3’

AR-Reverse: 5’-ACACCGACACTGCCTTACACAAC-3’

FOXA1-Forward: 5’- CTCGTACATCTCGCTCATCACCATG -3’

FOXA1-Reverse: 5’- CGCAAGTAGCAGCCGTTCTCG -3’

GATA2-Forward: 5’- CACTGACGGAGAGCATGAAGATGG -3’

GATA2-Reverse: 5’- GGCTTGATGAGTGGTCGGTTCTG -3’

SLC2A3-Forward: 5’-TGCCATCACAGTTGCTACAATCGG -3’

SLC2A3-Reverse: 5’-GCCACCAGTGACAGCCAACAG -3’

DHCR24-Forward: 5’-CCTCTTCCTCCTGCCGCTCTC -3’

DHCR24-Reverse: 5’-GGTGTCCACTTCCAGAATGTCCATC -3’

TDO2-Forward: 5’-GAGACGATGACAGCCTTGGACTTC-3’

TDO2-Reverse: 5’-CCAGCCATGCCTCCACTAATTCC-3’

GR-Forward: 5’-CATGTTAGGAGGGCGGCAAGTG-3’

GR-Reverse: 5’-TGGCTCTTCAGACCGTCCTTAGG-3’

KLK2-Forward: 5’- CTTCCCACACCCGCTCTACAATATG -3’

KLK2-Reverse: 5’- TTGGACAGGAGATGGAGGCTCAC -3’

FKBP5-Forward: 5’- TATGGCTCGGCTGGCAGTCTC -3’

FKBP5-Reverse: 5’- GGAATGTCGTGGTCTTCTCCTTCG -3’

CYP1A1-Forward: 5’-CTGTCAAGGATGAGCCAGCAGTATG-3’

CYP1A1-Reverse: 5’-CCAGGTAGCAGGAGGTTGAGGAG-3’

CYP1B1-Forward: 5’-GACGCCTTTATCCTCTCTGCG-3’

CYP1B1-Reverse: 5’-ACGACCTGATCCAATTCTGCC-3’

GAPDH-Forward: 5’-GTGTCGCTGTTGAAGTCAGAGGAG-3’

GAPDH-Reverse: 5’-CAAGGCTGTGGGCAAGGTCATC-3’

ChIP-qPCR assay

The following primers were used:

L1PA5#1(TDO2 transposons GR binding site):

5’-CTGAGAGATTTTGTCAACACC-3’ (forward)

and 5’- GGTCTTTACAATTTGGCATG-3’ (reverse).

L1PA5#2(TDO2 transposons GR binding site):

5’- CTTTACAGACAAGCAAATGCTG-3’ (forward)

and 5’- TCTAGCCTCGAAGGTCTTTAC -3’ (reverse).

TDO2 8th intron AR binding site:

5’- GTGGGTGAGTTCTTTCATAC-3’ (forward)

and 5’- GGGATGCGTTACAATAAAAG -3’ (reverse).

GRE-1 (TDO2 promotor GR binding site):

5’- CGTACCCAGTTGAACAAC-3’ (forward)

and 5’- CACACTGGATAGGGACCTTA -3’ (reverse).

GRE-2 (TDO2 promotor GR binding site):

5’- GTAGGCAATTTCCAGGATC-3’ (forward)

and 5’- AATCTTCAAGATCCTGACCC -3’ (reverse).

GRE-3 (TDO2 promotor GR binding site):

5’- GCTAAGGATAAGGCCATGAAG-3’ (forward)

and 5’- GCCCATAATTCTATACAGTGTCAGTG -3’ (reverse).

Supplementary Fig. S1

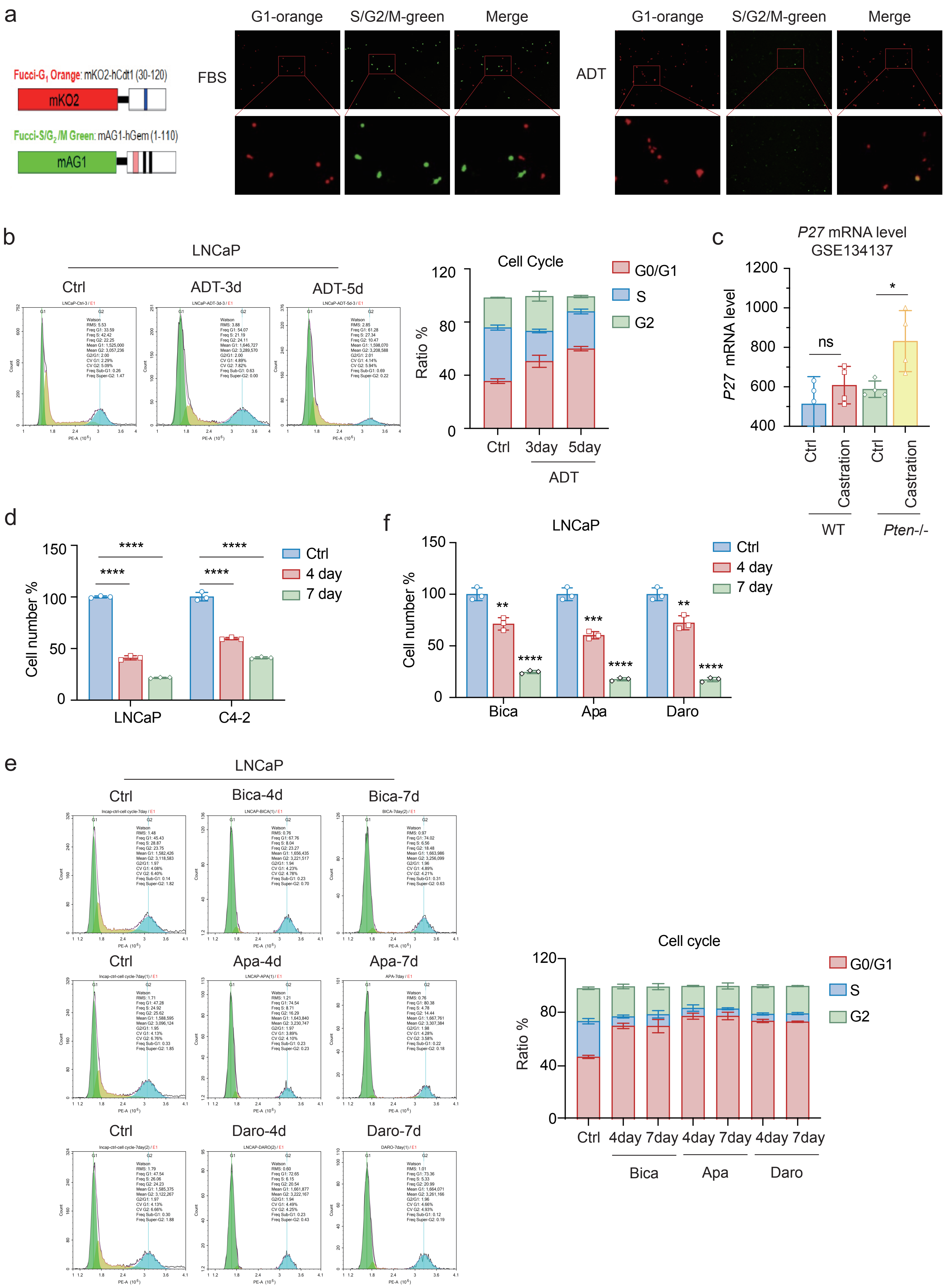

Supplementary Fig. S2

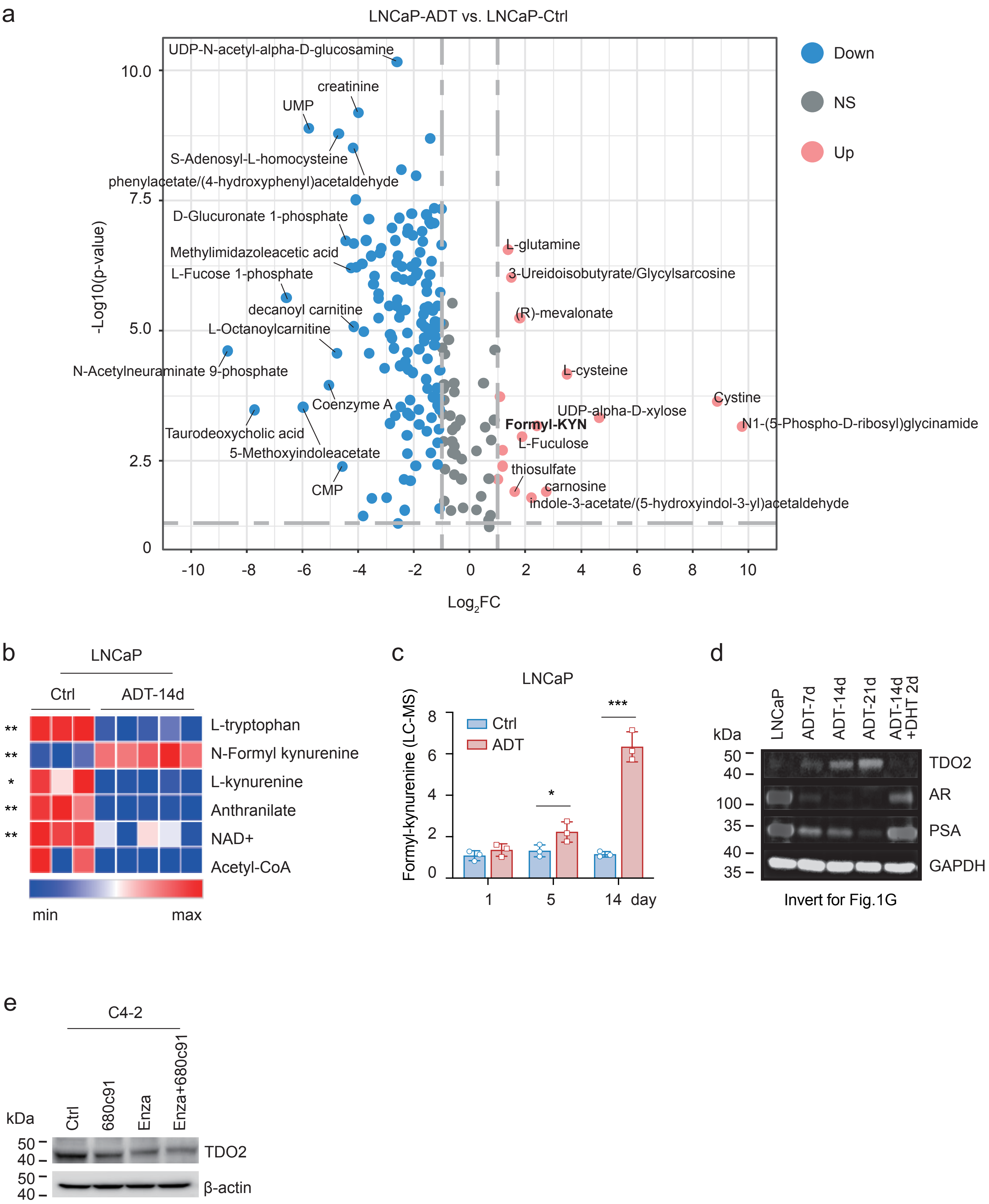

Supplementary Fig. S3

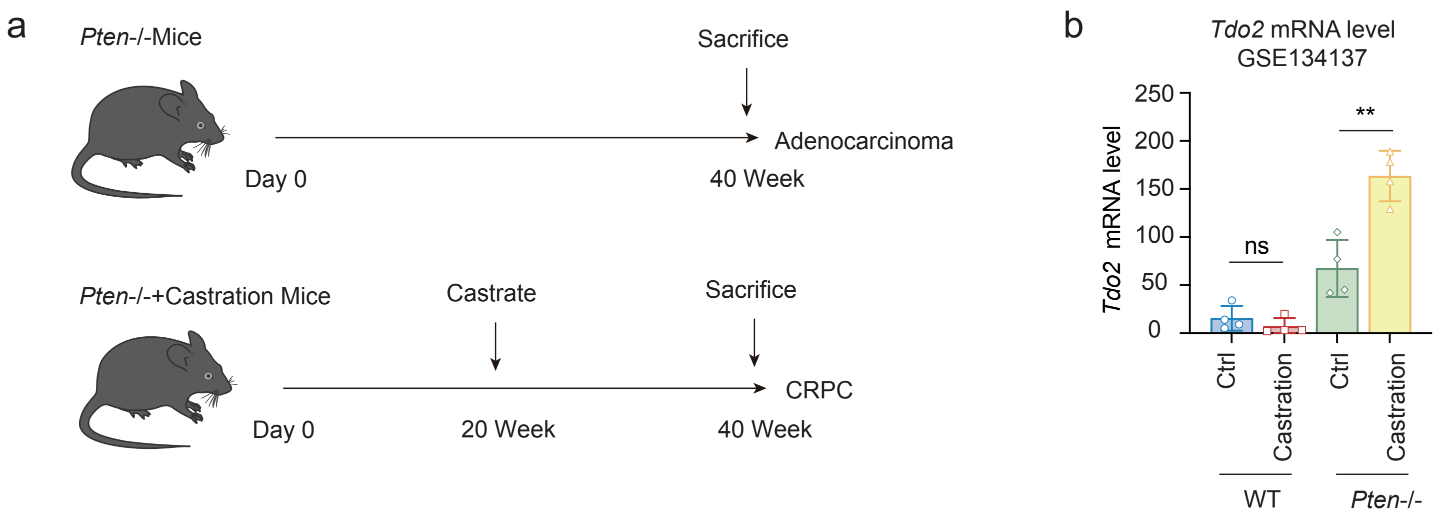

Supplementary Fig. S4

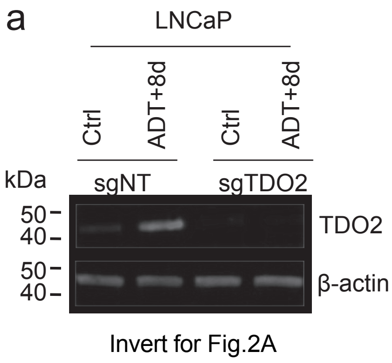

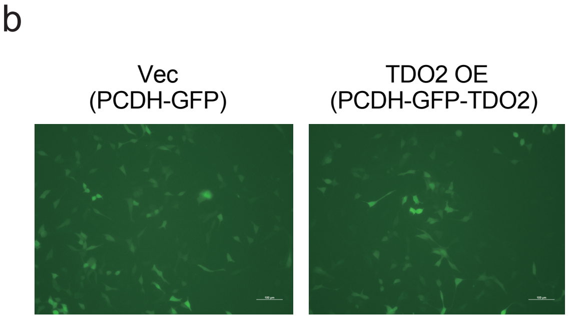

Supplementary Fig. S5

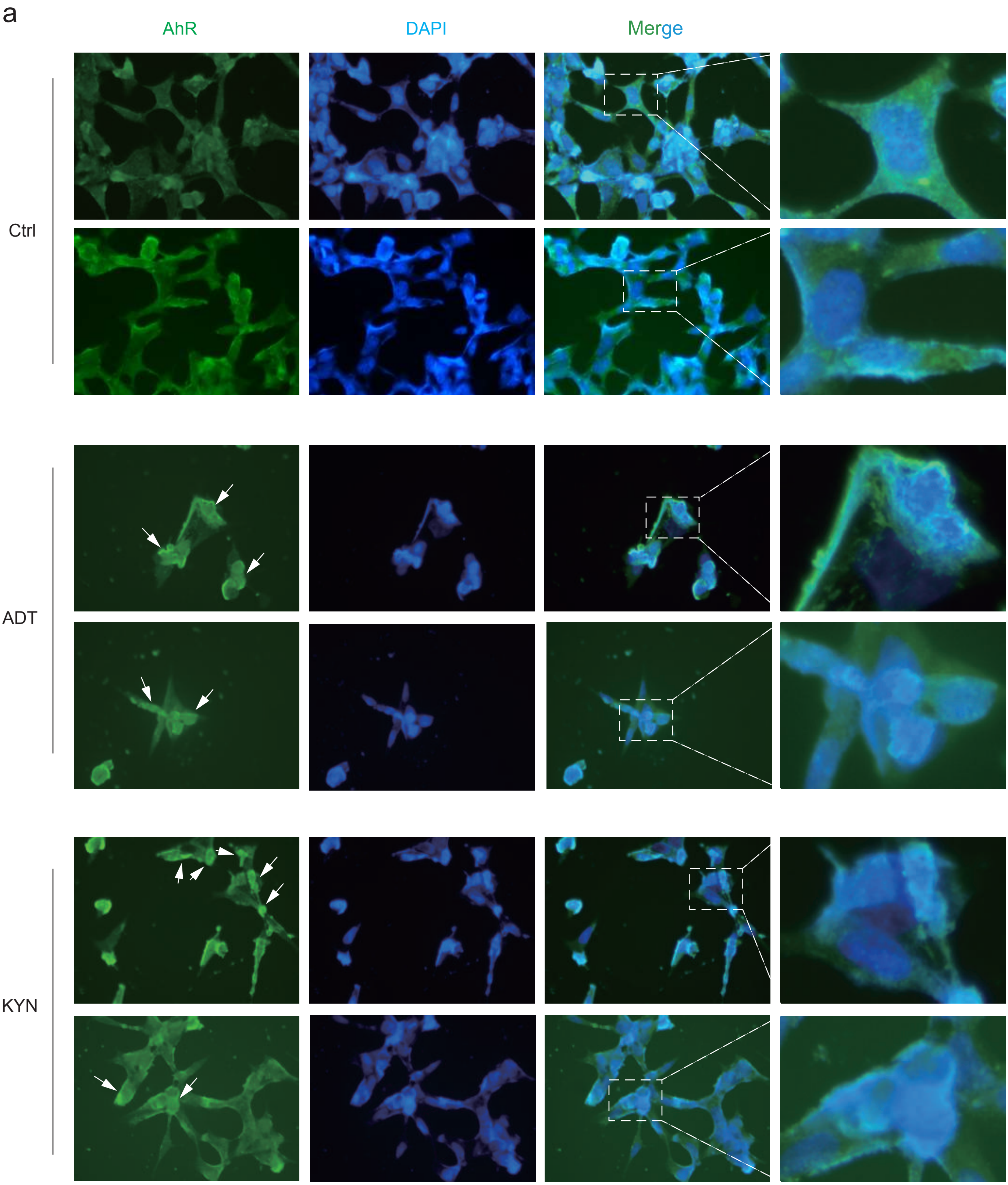

Supplementary Fig. S6

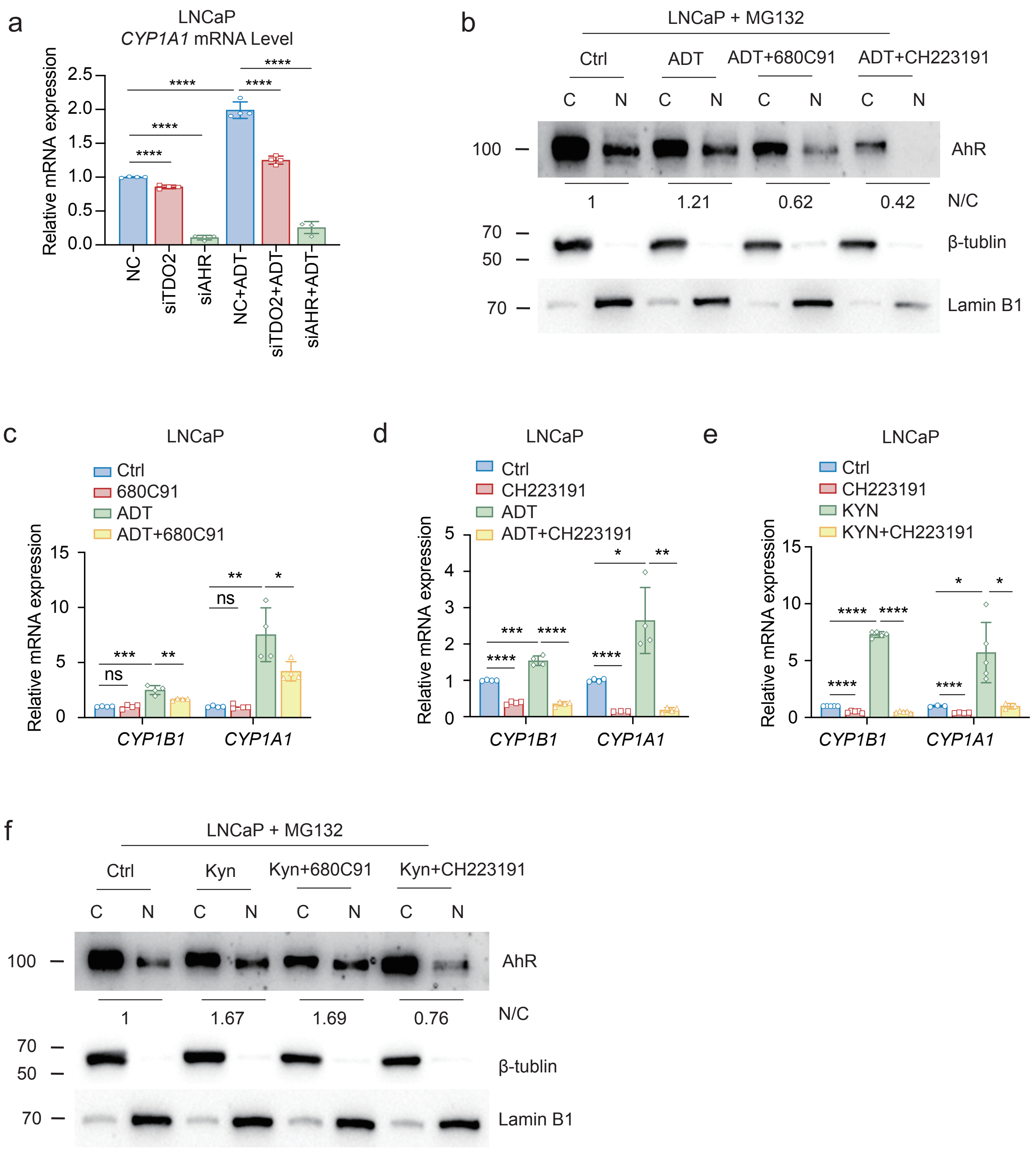

Supplementary Fig. S7

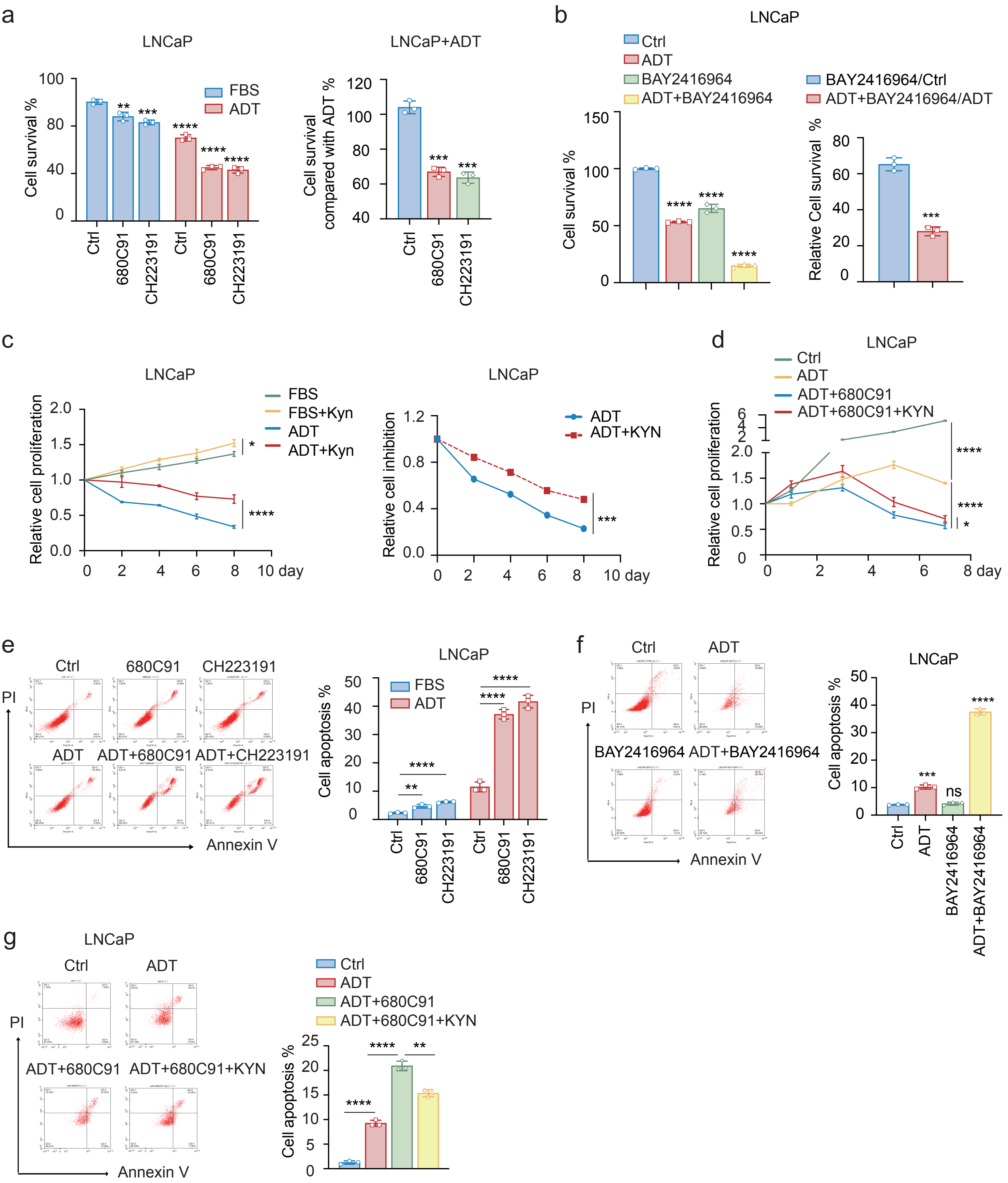

Supplementary Fig. S8

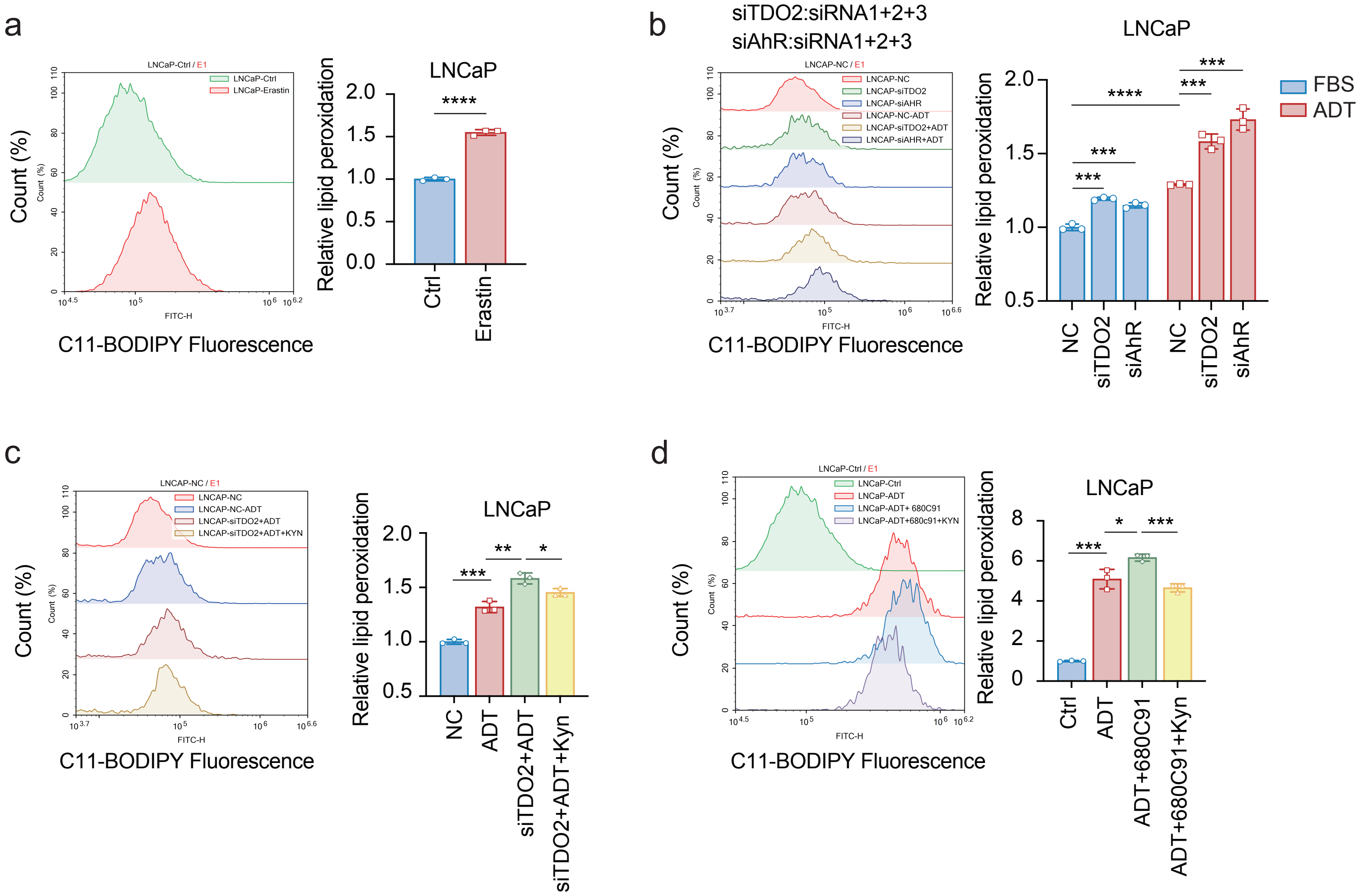

Supplementary Fig. S9

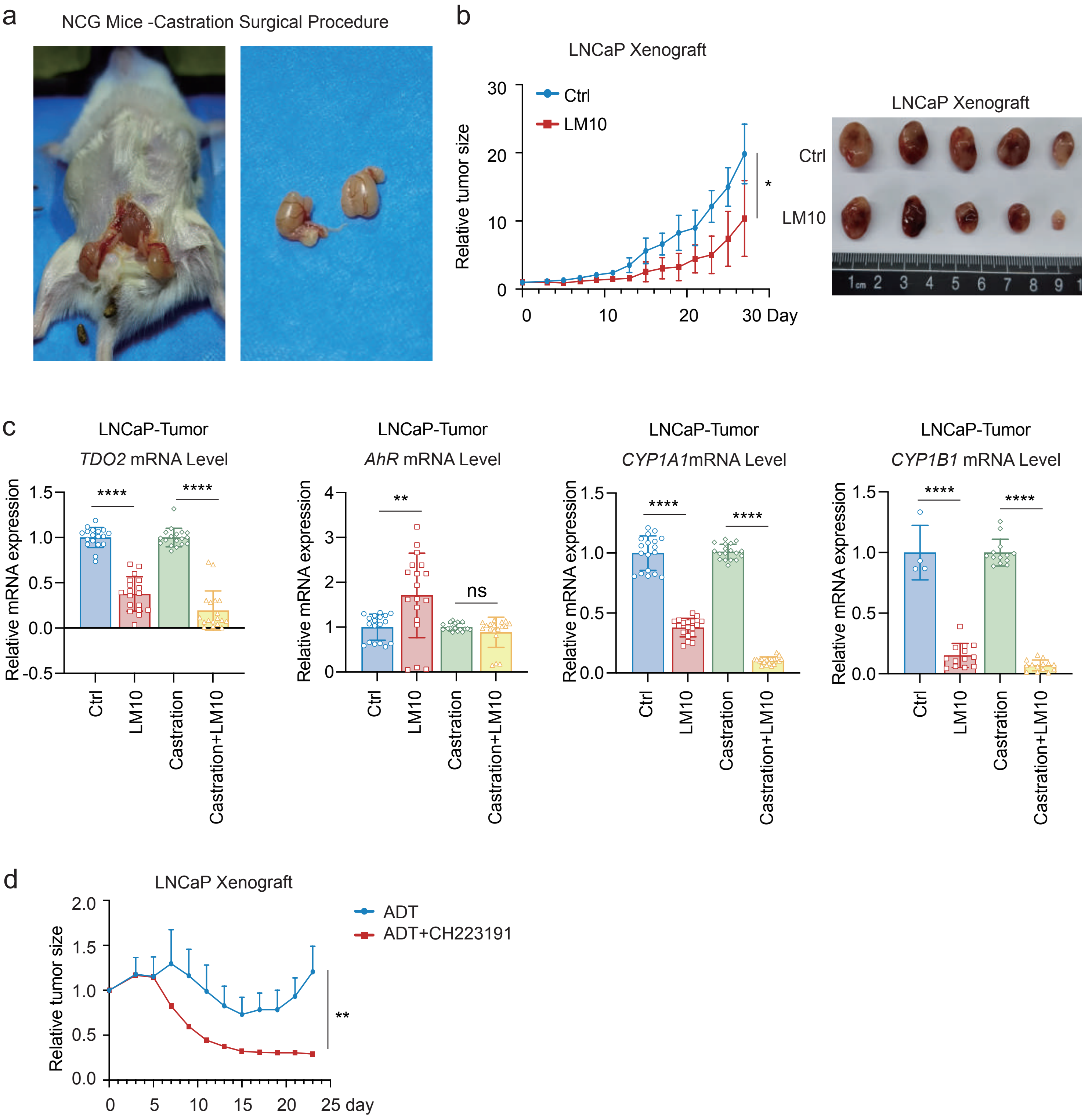

Supplementary Fig. S10

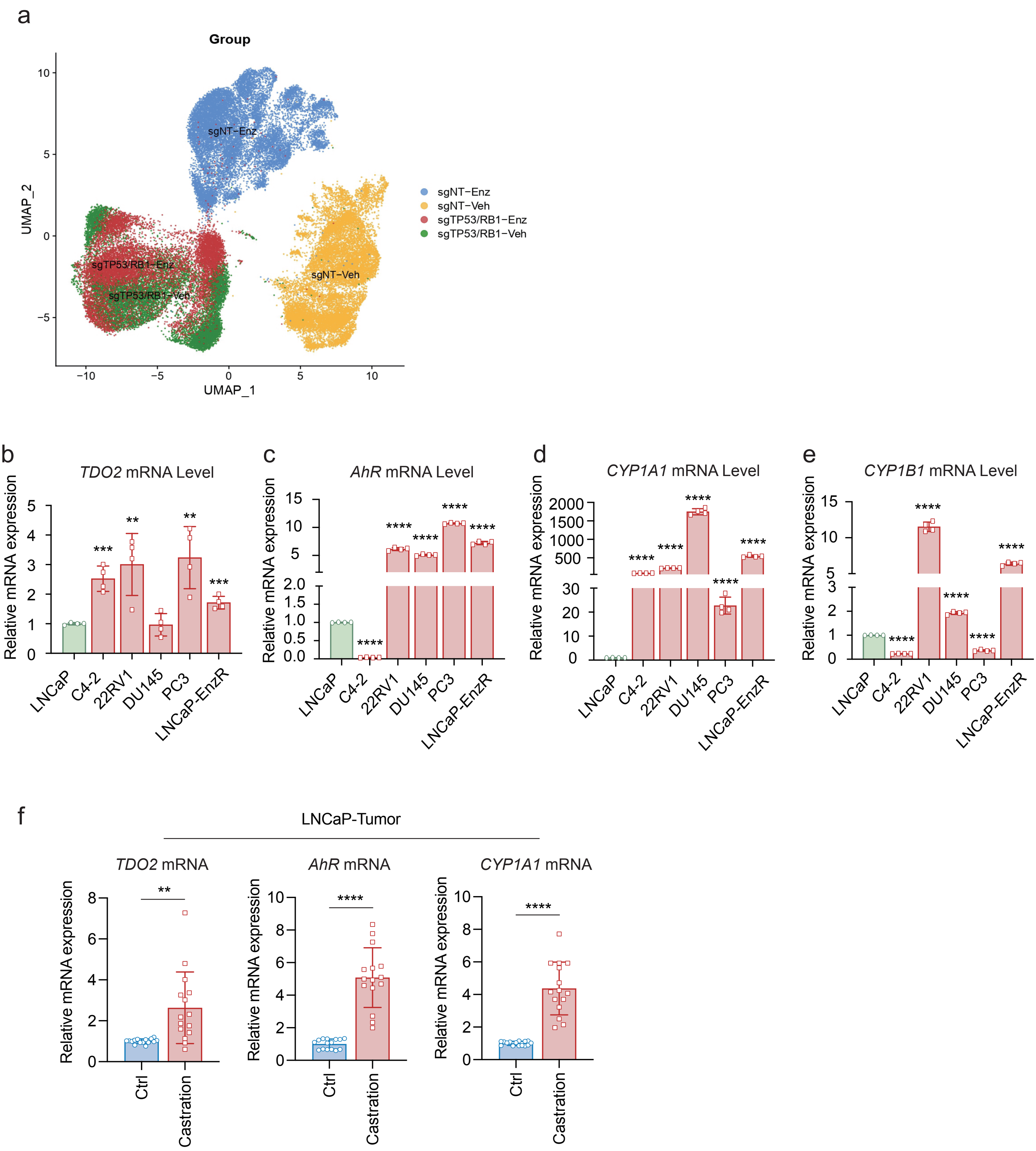

Supplementary Fig. S11

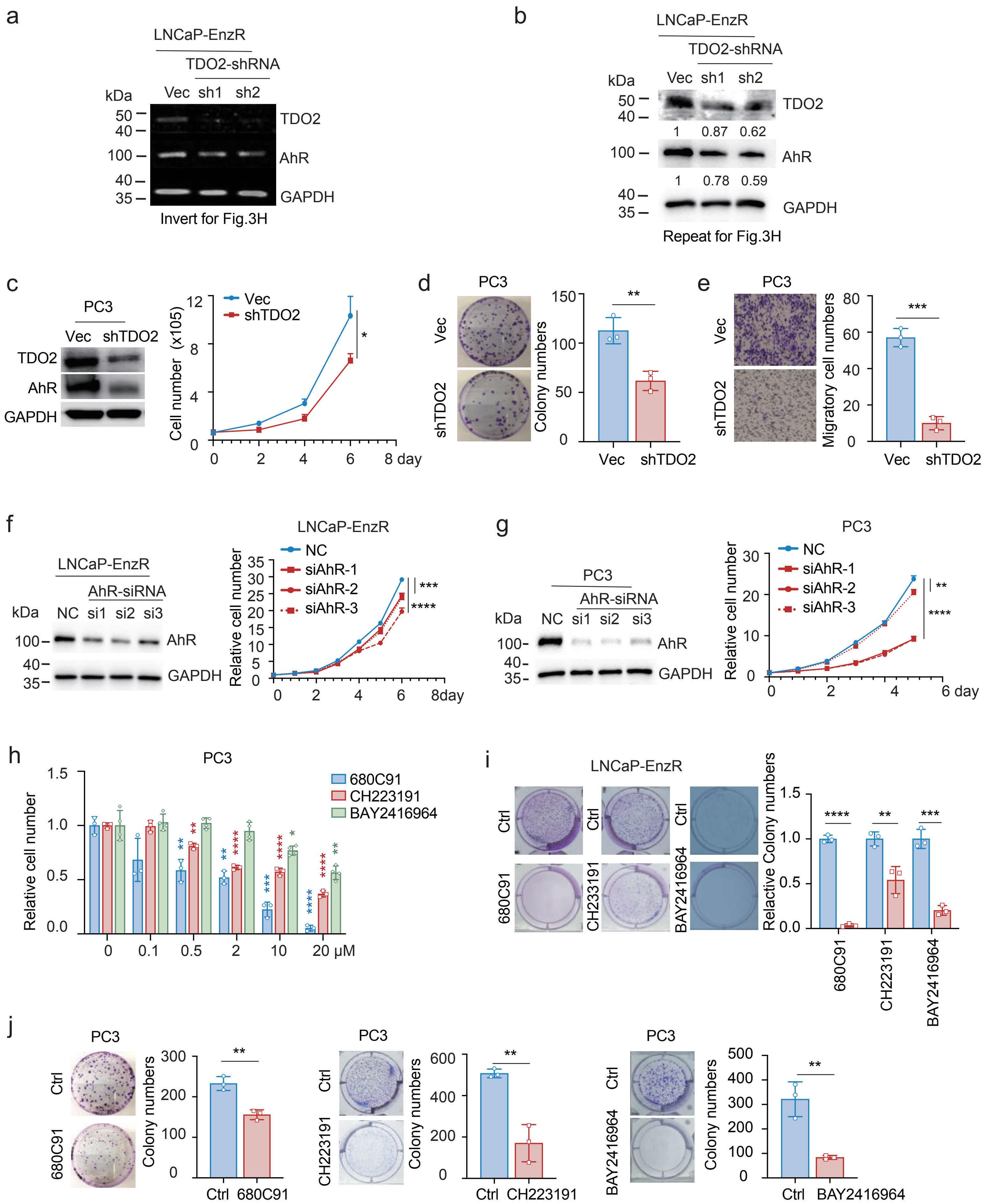

Supplementary Fig. S12

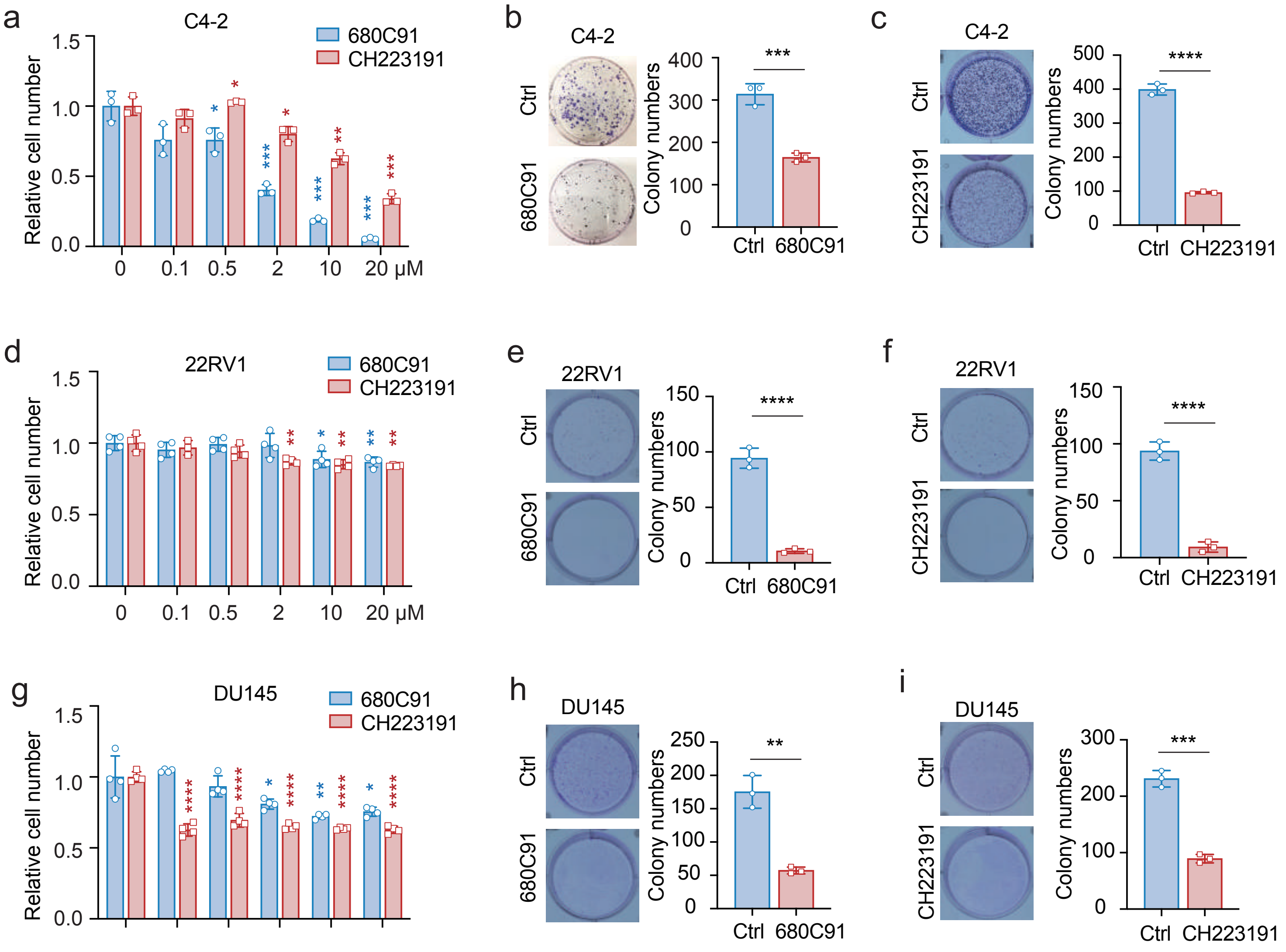

Supplementary Fig. S13

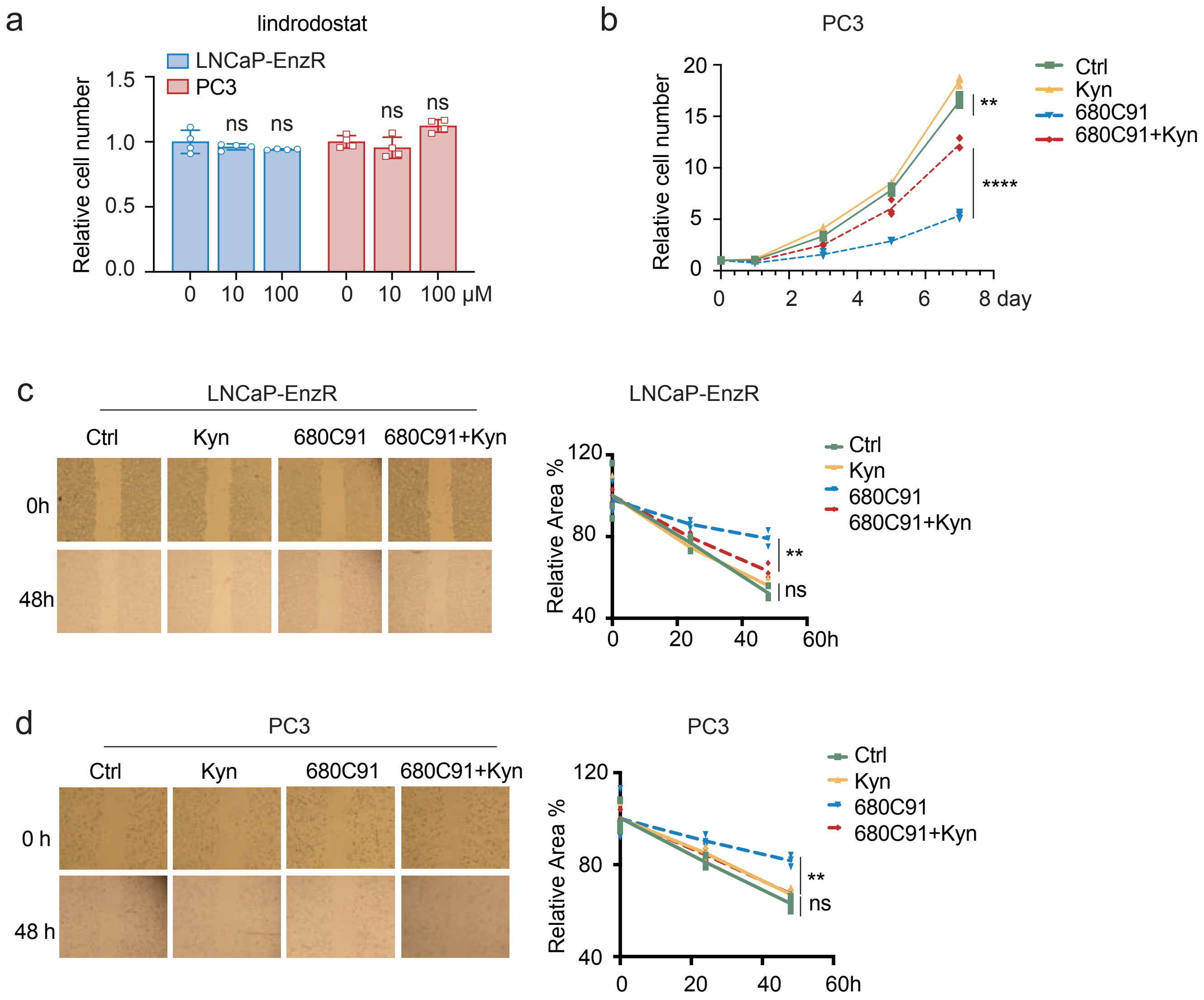

Supplementary Fig. S14

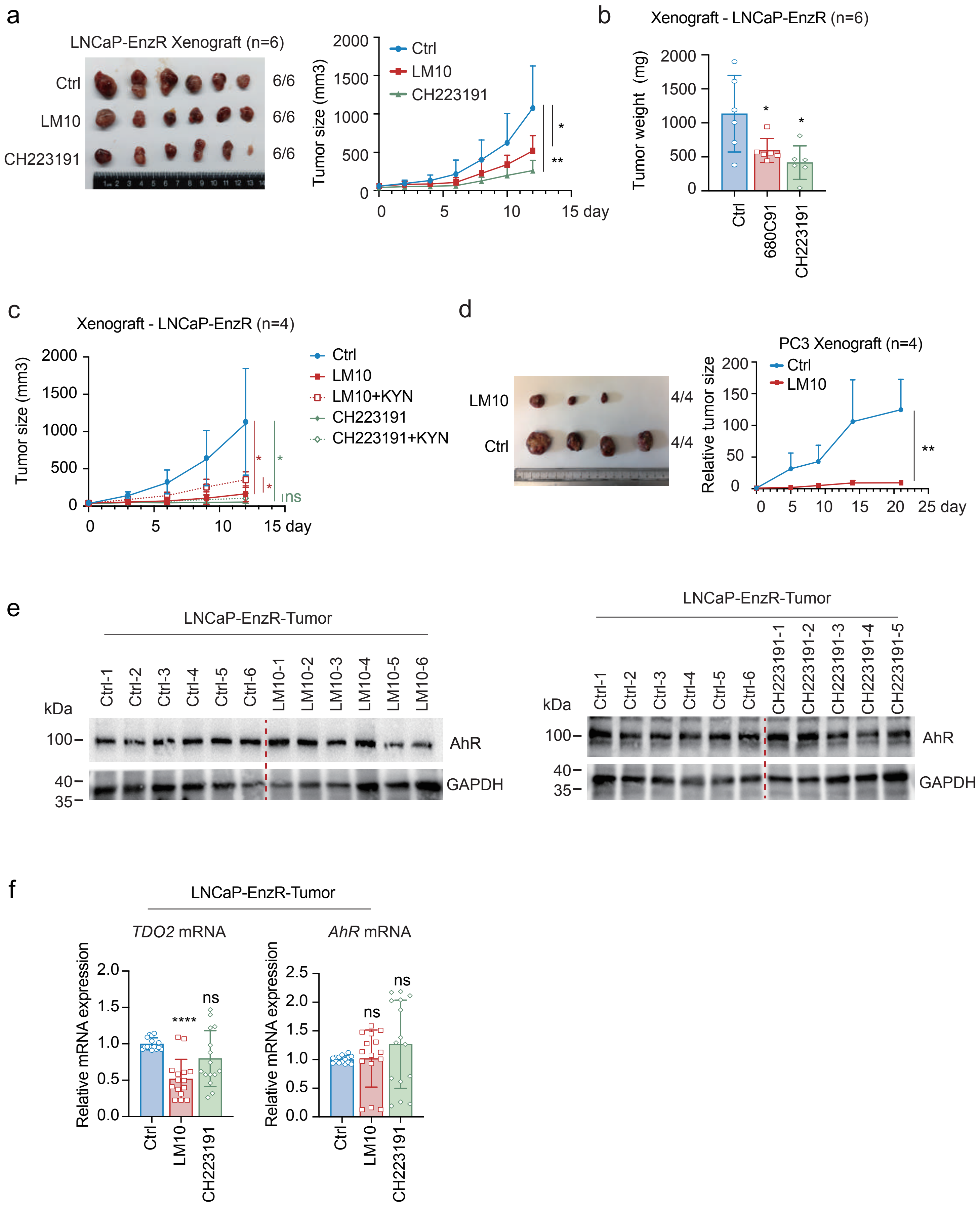

Supplementary Fig. S15

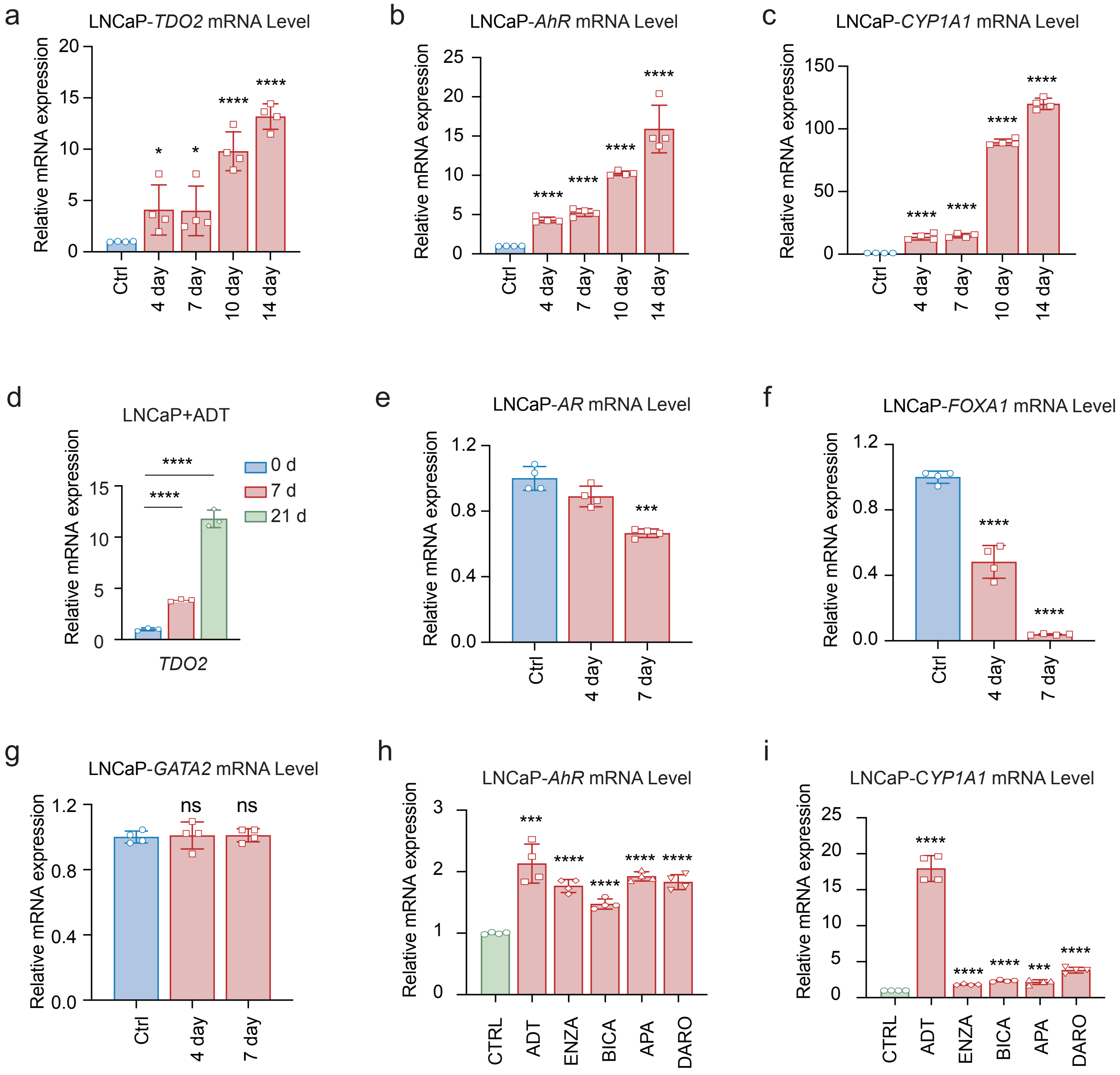

Supplementary Fig. S16

a

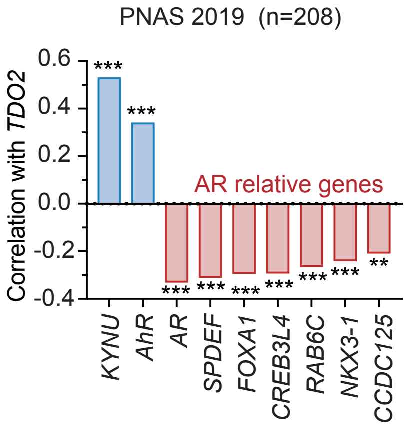

b

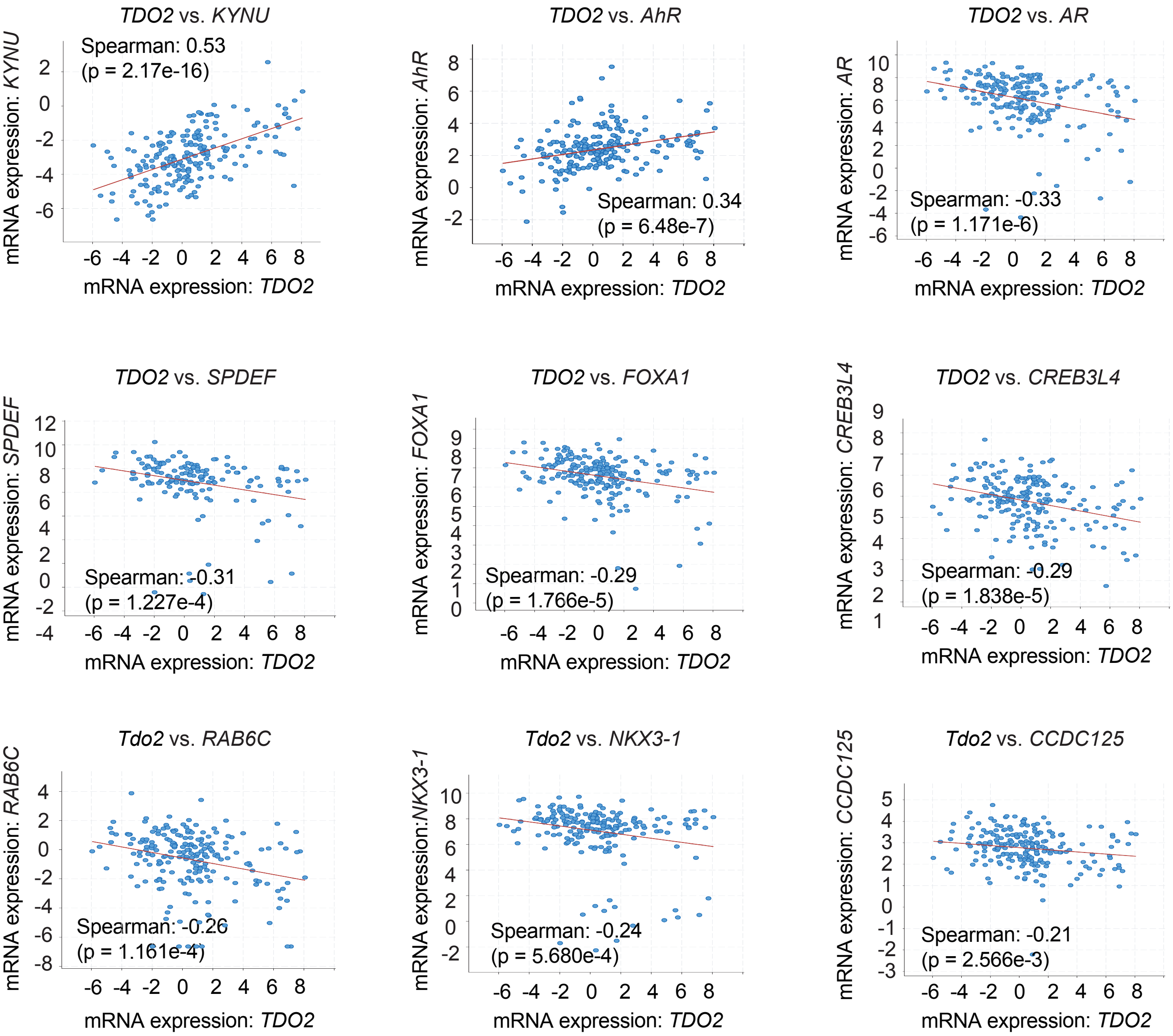

Supplementary Fig. S17

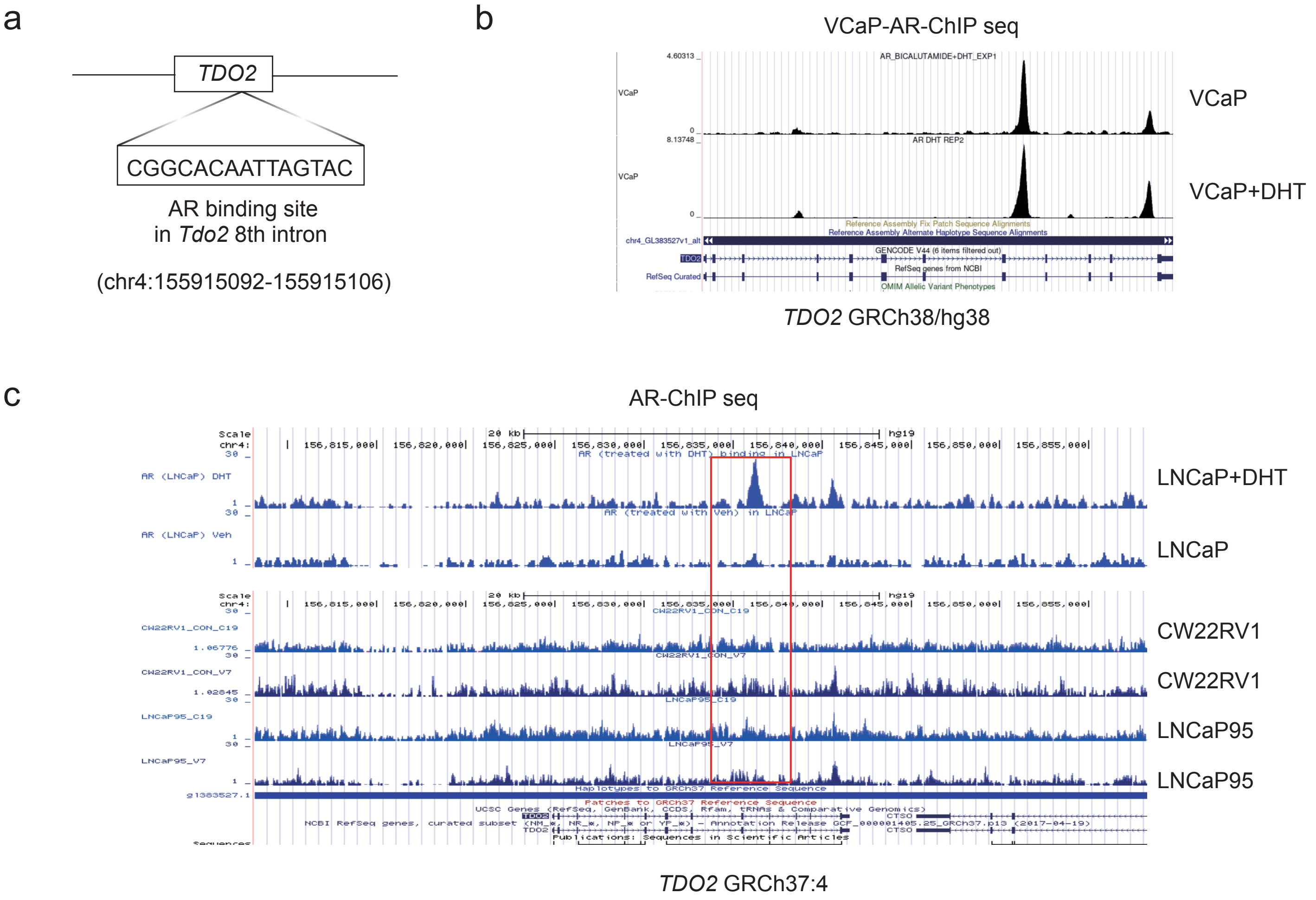

d

Factors predicted within a dissimilarity margin less or equal than 1%

0 TFIID [T00820] 1 GR–beta [T01920] 2 GR–alpha [T00337] 3 PR B [T00696] 4 PR A [T01661] 5 AP–2alphaA [T00035] 6 C/EBPbeta [T00581] 7 ER–alpha [T00261]
8 c–Ets–1 [T00112] 9 STAT4 [T01577] 10 YY1 [T00915] 11 POU2F1 [T00641] 12 AR [T00040] 13 TFII–I [T00824] 14 GATA–1 [T00306] 15 FOXP3 [T04280]
16 RXR–alpha [T01345] 17 HNF–1A [T00368] 18 Elk–1 [T00250] 19 NF–AT2 [T01945] 20 TBP [T00794] 21 TCF–4 [T02918] 22 HNF–3alpha [T02512] 23 AP–1 [T00029]
24 c–Jun [T00133] 25 RAR–beta [T00721]

Data from: http://alggen.lsi.upc.es/cgi-bin/promo_v3/promo/promoinit.cgi?dirDB=TF_8.3

Supplementary Fig. S18

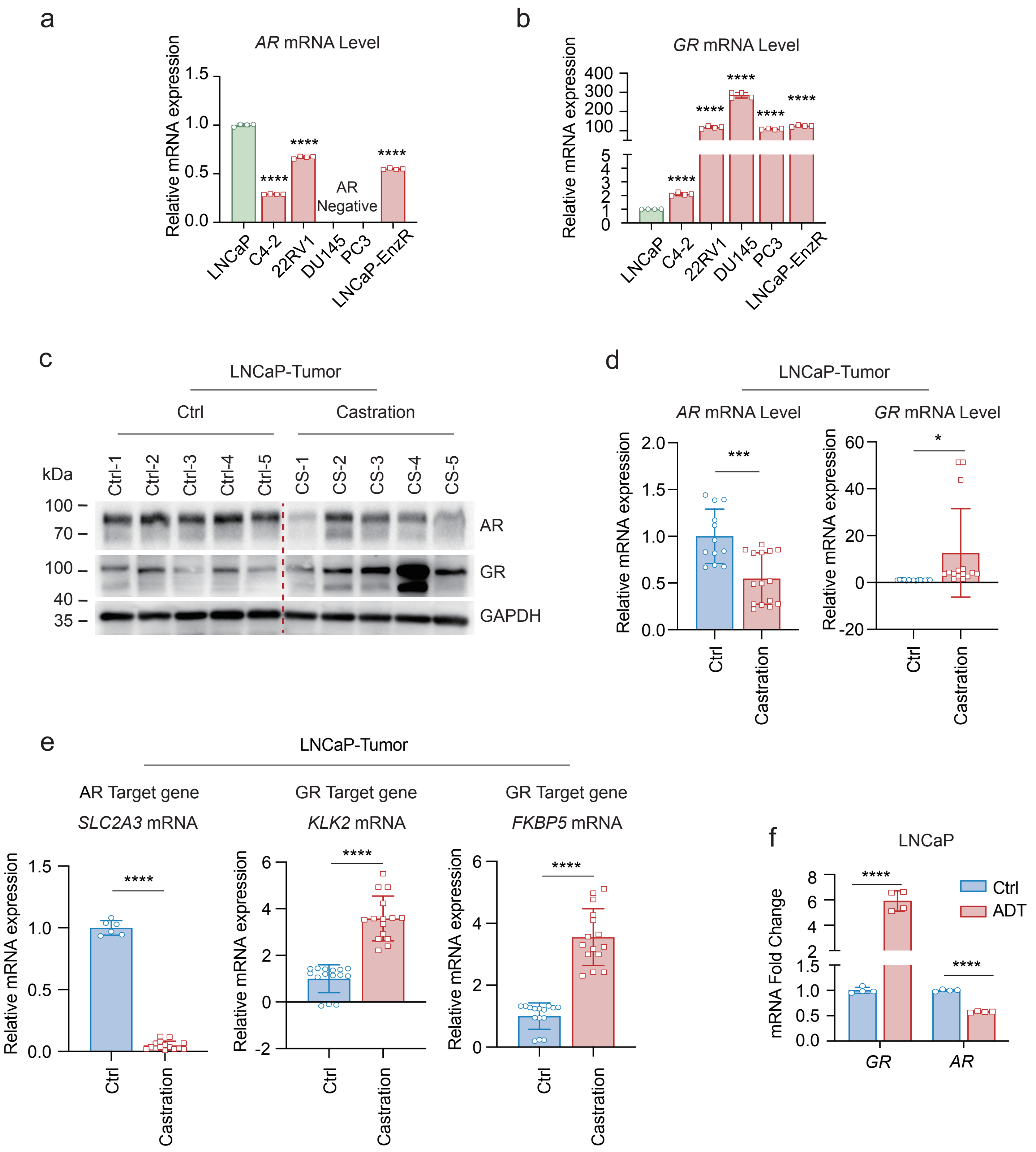

Supplementary Fig. S19

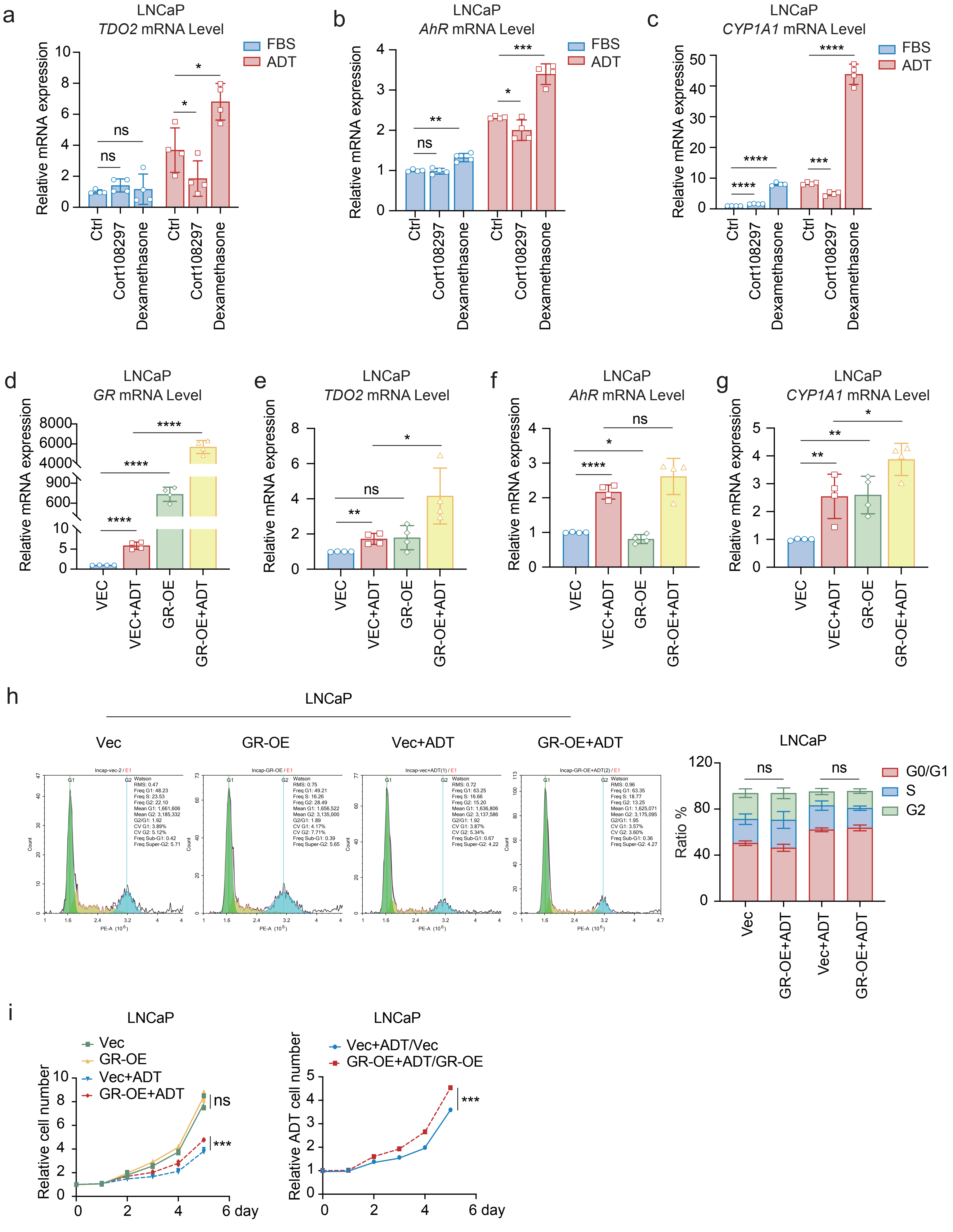

Supplementary Fig. S20

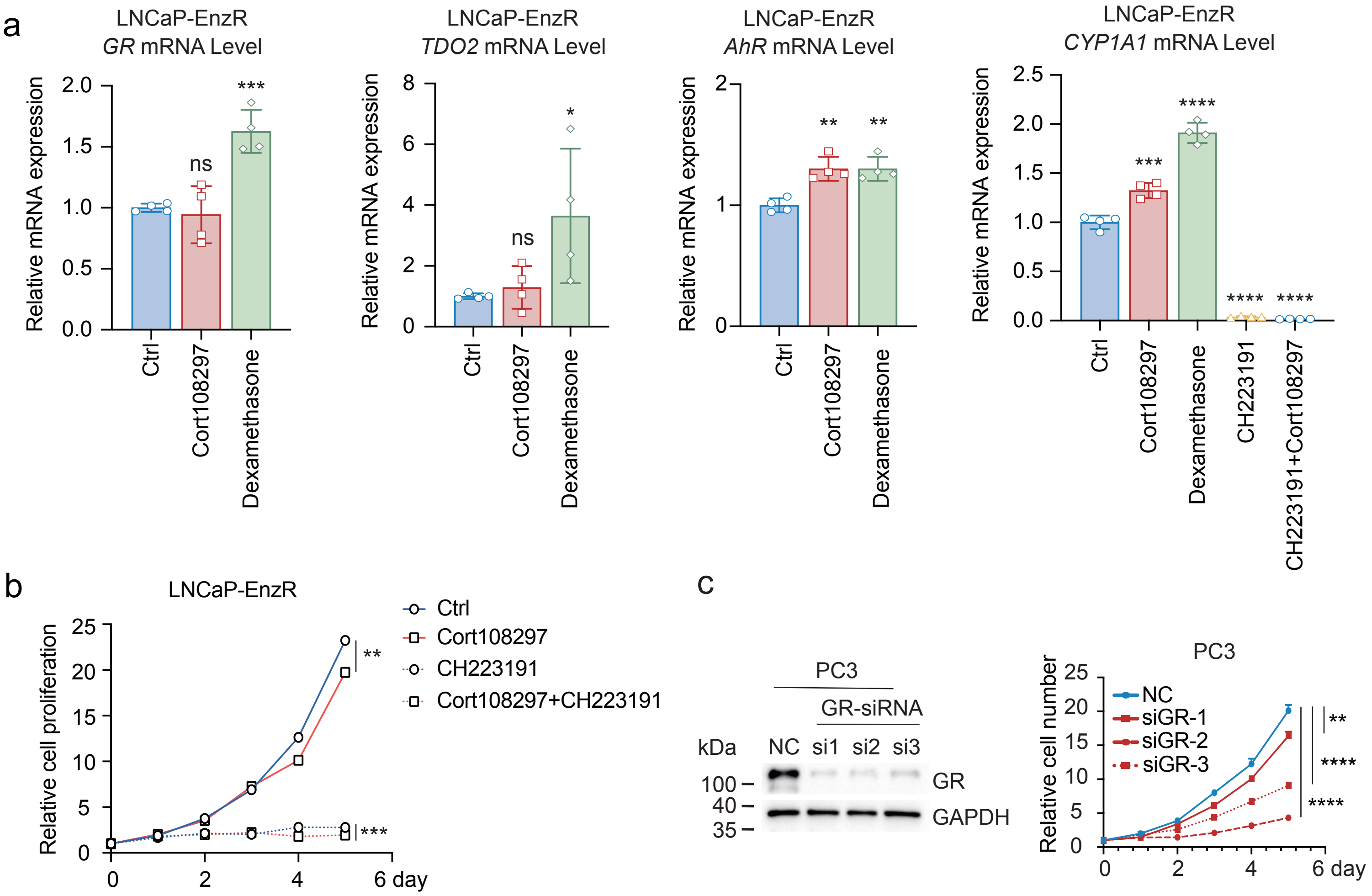

Supplementary Fig. S21

a

L1PA5	4837 bp	155892568—155897405
Peak1	201 bp	155893197—155893397
		（The GR site is in the 61st bp ）
Peak2	184 bp	155894370—155894553
GR binding site	17bp	155893257—155893274

b

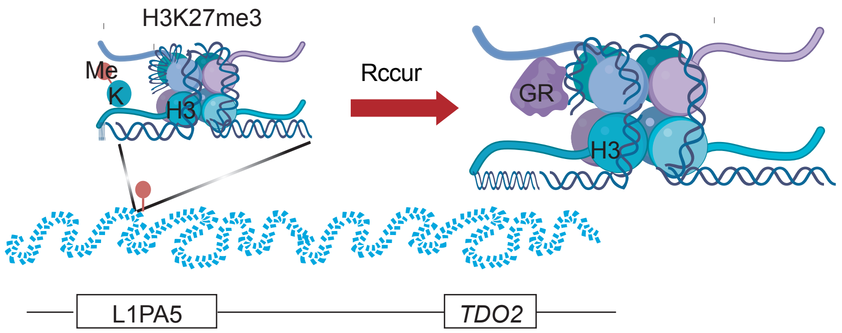

c

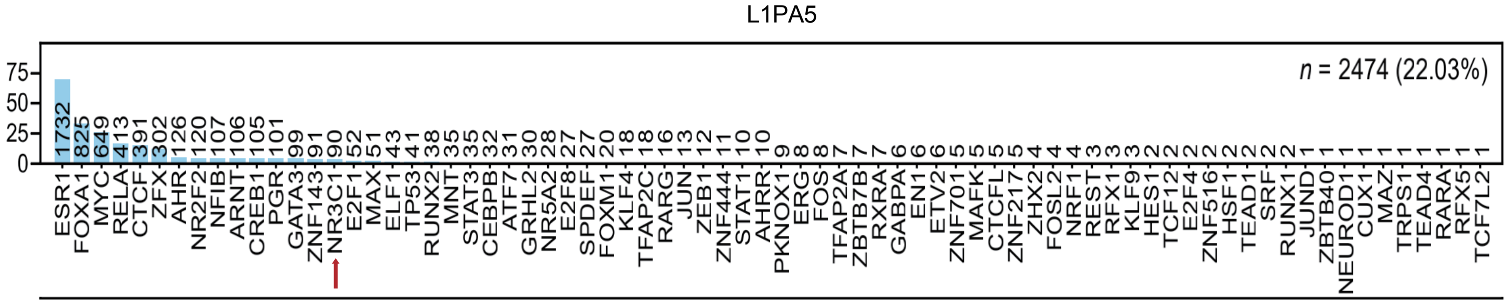

data from: Int J Mol Sci. 2021 May 25;22(11):5625. doi: 10.3390/ijms22115625.

Supplementary Fig. S22

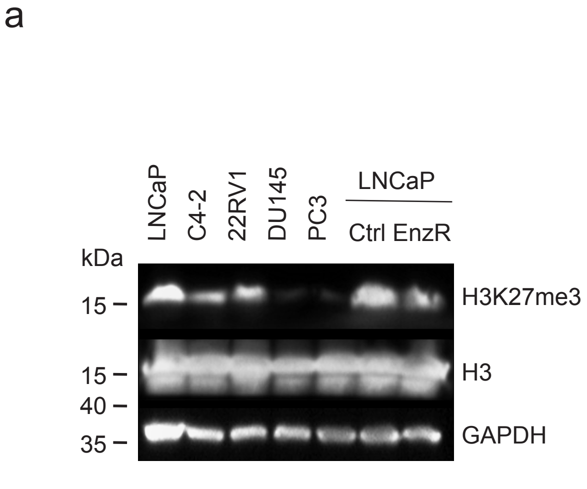

Invert for Fig.4N

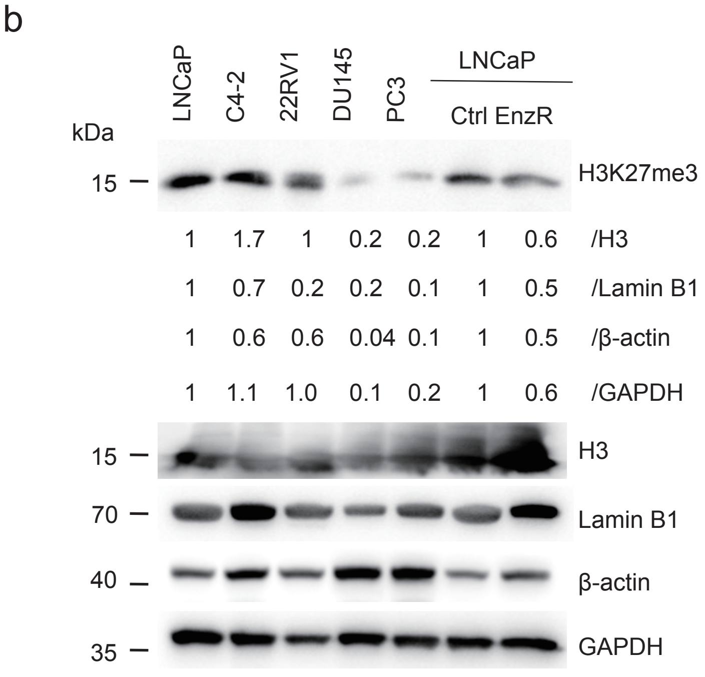

Repeat for Fig.4N

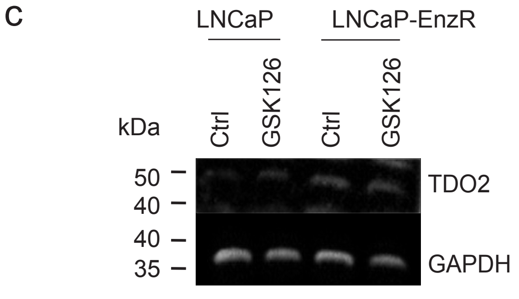

Invert for Fig.4O

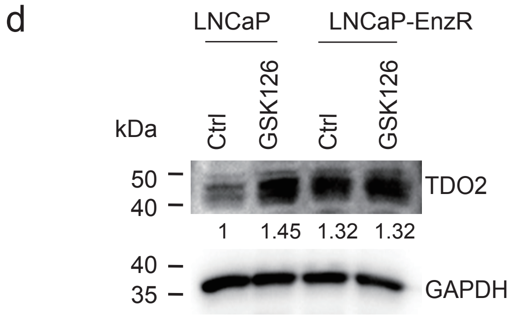

Repeat for Fig.4O

Supplementary Fig. S23

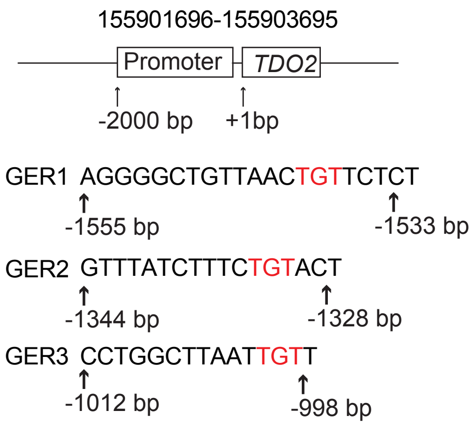

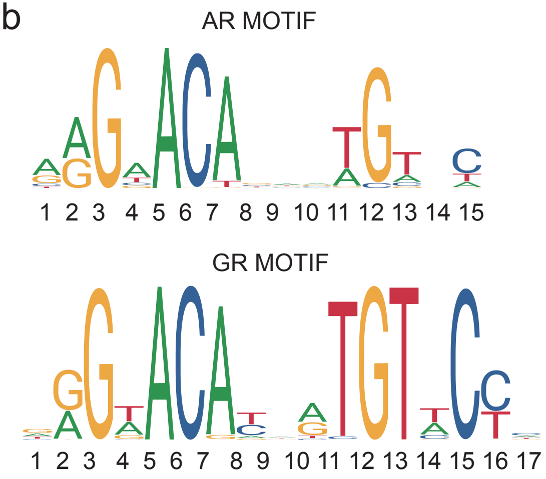

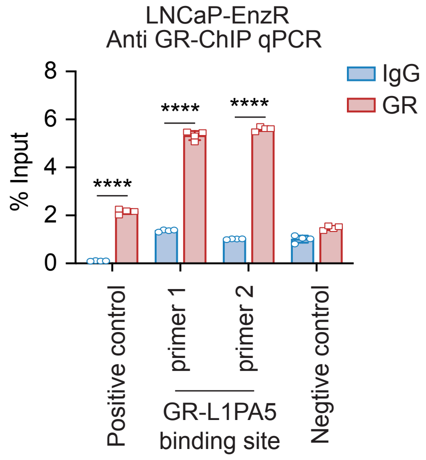

Supplementary Fig. S24

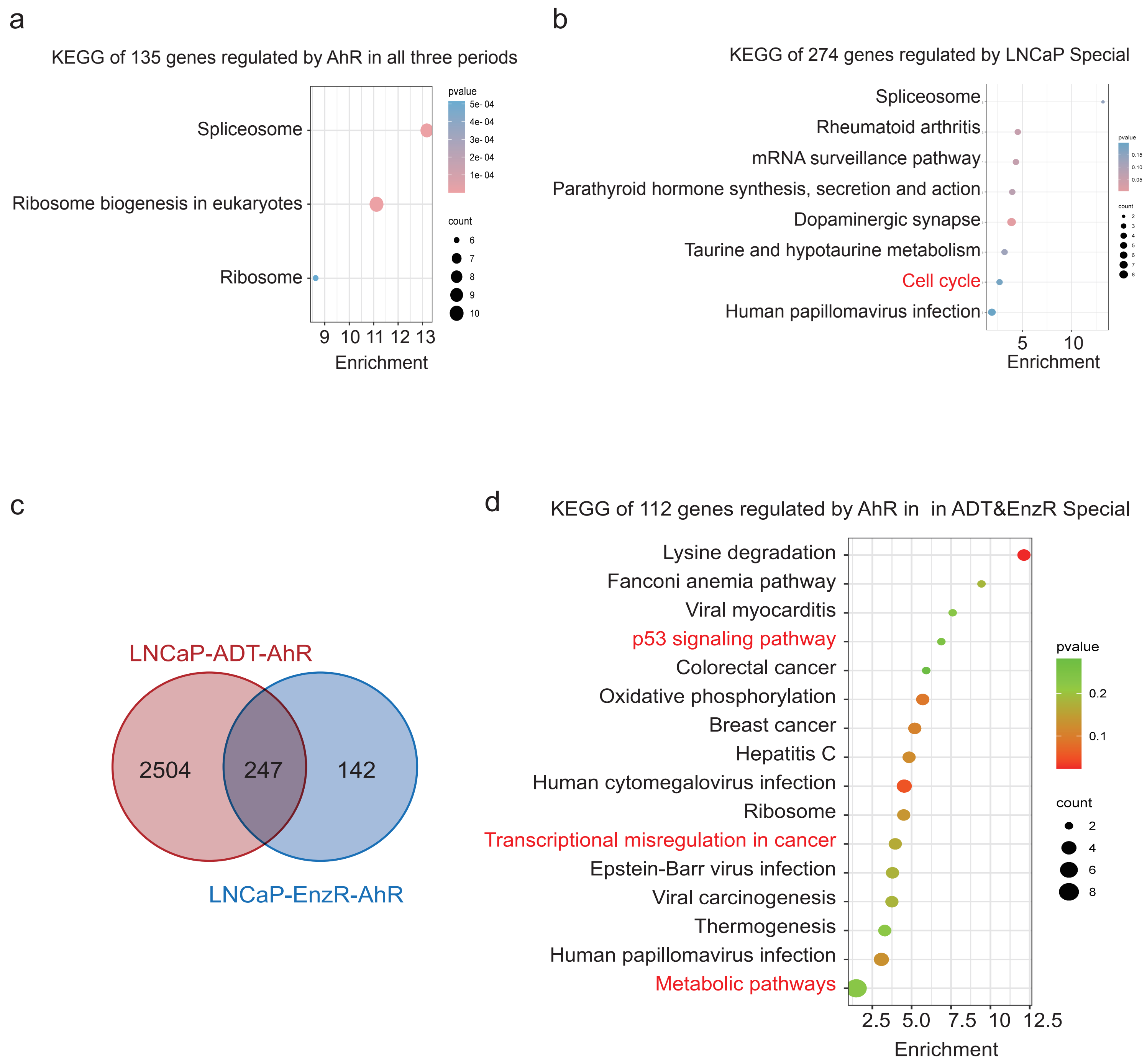

Supplementary Fig. S25

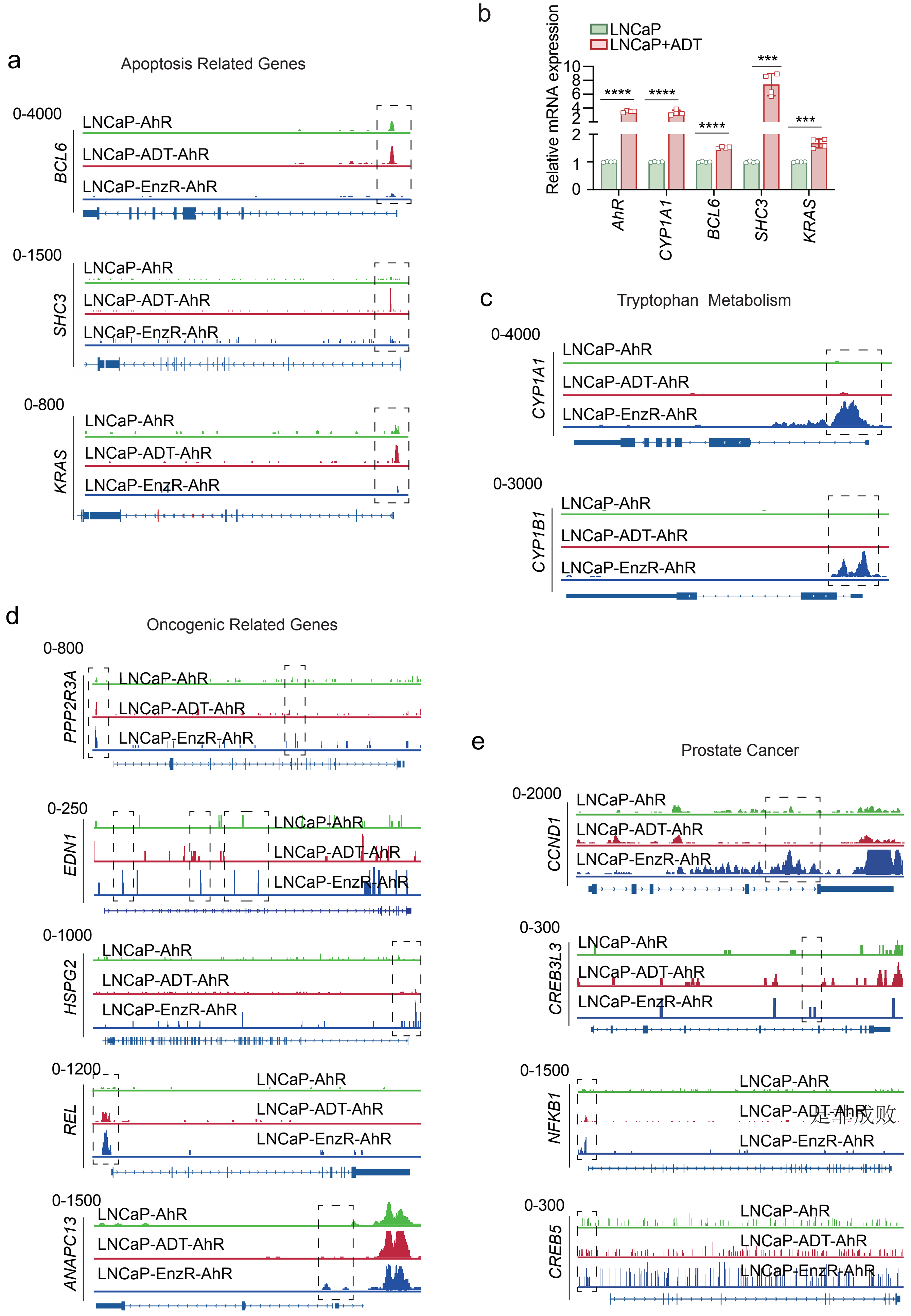

Supplementary Figure legends

Supplementary Fig. S1

(a) The FUCCI system was used to label the cell cycle, and Orange linked the human *Cdt1* gene, which is mainly expressed in the G1 phase of the cell cycle. Green link human *Gem* gene, mainly expressed in the S/G2/M phase of the cell cycle. **(b)** The proportion of cell cycle in LNCaP cell lines under different treatment time of ADT was examined. **(c)** *P27* mRNA levels from RNA sequencing of wild-type control and *Pten* conditional knockout (*Pten*-/ -cKO) mouse prostate tumor tissues after sham or castration (data from GSE134137). **(d)** In LNCaP and C4-2 cells, enzalutamide was added, and cell number was measured at 0, 4, and 7 days. **(e)** The cell cycle ratio of LNCaP cells treated with Bicalutamide, Apalutamide, and Darolutamide for 0, 4 and 7 days. **(f)** In LNCaP cells, Bicalutamide, Apalutamide, and Darolutamide was added, and cell number was measured at 0, 4, and 7 days. *, $P < 0.05$, **, $P < 0.01$, ***, $P < 0.001$, and ****, $P < 0.0001$.

Supplementary Fig. S2

(a) Volcano map (day 14) shows that ADT treatment significantly decreased the levels of most metabolites but increased the levels of a few metabolites. **(b)** Metabolic mass spectrometry was performed on LNCaP cells cultured at days 1, 5, and 14 in medium containing normal FBS and activated stripped FBS (ADT). The heat map shows the changes in tryptophan metabolic pathways in the metabolomic analysis. **(c)** Changes in the relative concentration of Formyl-kynurenine in LNCaP cell lines at different ADT treatment times from the metabolomic analysis. **(d)** Perform image inversion on Fig1G using ImageJ. **(e)** The C4-2 cell line was treated with 680C91 or enzalutamide or cotreatment for 72h, and the protein expression level of TDO2 was determined by Western blot. *, $P < 0.05$, **, $P < 0.01$, ***, $P < 0.001$, and ****, $P < 0.0001$.

Supplementary Fig. S3

(a) The progression of prostate cancer was reproduced using wild-type control and Pten conditional knockout mice (*Pten*-/ -cKO). The prostate tumor tissue was taken for immunohistochemical staining. **(b)** *tdo2* mRNA levels from RNA sequencing of wild-type control and *Pten* conditional knockout (*Pten* -/ -cKO) mice after sham or castration (data from GSE134137). *, P < 0.05, **, P < 0.01, ***, P < 0.001, and ****, P < 0.0001.

Supplementary Fig. S4

(a) Perform image inversion on Fig2A using ImageJ. **(b)** PCDH-GFP or pcDH-GFP-TDO2 lentivirus was transfected in LNCaP cells and fluorescence was observed under a microscope. *, $P < 0.05$, **, $P < 0.01$, ***, $P < 0.001$, and ****, $P < 0.0001$.

Supplementary Fig. S5

(a) After conducting ADT or Kyn treatment on LNCaP for 6 hours (Each group was supplemented with 10uM MG132), immunofluorescence staining was performed to observe the distribution of AhR in the cytoplasm and nucleus under a fluorescence microscope. The green fluorescence in the figure represents AhR, while the blue fluorescence represents DAPI. *, $P < 0.05$, **, $P < 0.01$, ***, $P < 0.001$, and ****, $P < 0.0001$.

Supplementary Fig. S6

(a) The mRNA levels of *CYP1A1* were detected after the addition of siTDO2 or siAhR to LNCaP cells, with or without ADT treatment. **(b)** Perform ADT, ADT+680C91, and ADT+CH223191 treatment on LNCaP for 6 hours. Conduct nuclear-cytoplasmic separation and WB testing of AhR distribution in the cytoplasm and nucleus. **(c)** *Cyp1a1* and *Cyp1b1* mRNA levels were measured after the addition of TDO2 inhibitor 680C91, ADT or in combination (48h) in LNCaP cells. **(d)** *CYP1A1* and *CYP1B1* mRNA levels were measured after the addition of AhR inhibitor CH223191 or ADT or in combination (24h) in LNCaP cells. **(e)** *CYP1A1* and *CYP1B1* mRNA levels were measured after the addition of AhR inhibitor CH223191 or Kyn or in combination (24h) in LNCaP cells. **(f)** Perform Kyn, Kyn +680C91, and Kyn +CH223191 treatment on LNCaP for 6 hours. Conduct nuclear-cytoplasmic separation and WB testing of AhR distribution in the cytoplasm and nucleus. *, $P < 0.05$, **, $P < 0.01$, ***, $P < 0.001$, and ****, $P < 0.0001$.

Supplementary Fig. S7

(a) AhR inhibitor CH223191 or TDO2 inhibitor 680C91 was used to examine cell survival in LNCaP cells with or without ADT treatment (48h). **(b)** The cell survival of LNCaP cells treated with AhR inhibitor BAY2416964 combined with ADT was measured.**(c)** Kyn was added to ADT-treated LNCaP cells and the cell growth was measured on days 0, 2, 4, 6, and 8. **(d)** Kyn was added to LNCaP cells treated with ADT in combination with the TDO2 inhibitor 680C91 and cell proliferation was measured on days 0, 1, 3, 5, and 7. **(e)** Cell apoptosis was measured for LNCaP cells treated with or without ADT in combination with AhR inhibitor CH223191 or TDO2 inhibitor 680C91 (48h). ADT treated 5 days in advance. **(f)** The cell apoptosis of LNCaP cells treated with AhR inhibitor BAY2416964 combined with ADT was measured. **(g)** Kyn was added to LNCaP cells treated with ADT treatment in combination with the TDO2 inhibitor 680C91 and cell apoptosis was measured on day 5. *, $P < 0.05$, **, $P < 0.01$, ***, $P < 0.001$, and ****, $P < 0.0001$.

Supplementary Fig. S8

(a) The LNCaP cells were treated with the ferroptosis inducer Erastin to measure the levels of lipid peroxidation (Erastin serving as a positive control). **(b)** Transfection of siTDO2 or siAhR in LNCaP cells, with or without ADT treatment, to assess cellular lipid peroxidation levels. **(c)** Add Kyn to the transfected siTDO2 LNCaP cells in ADT treatment to detect the cellular lipid peroxidation levels. **(d)** The combination of Kyn and the TDO2 inhibitor 680C91 was added to LNCaP cells treated with ADT, and the cellular lipid peroxidation levels were measured. *, $P < 0.05$, **, $P < 0.01$, ***, $P < 0.001$, and ****, $P < 0.0001$.

Supplementary Fig. S9

(a) Procedure of castration surgery in NCG mice. **(b)** LNCaP cells were inoculated into the axillary subcutaneous tissue of NCG mice and grown for 3 weeks. Mice were treated with placebo or the TDO2 inhibitor LM10 (n=5). **(c)** RNA from LNCaP tumors was extracted, and the mRNA levels of *TDO2, AhR, CYP1A1* and *CYP1B1* were detected by RT-qPCR (n=5). **(d)** LNCaP cells were subcutaneously seeded into the axillary subcutaneous tissue of NCG mice and allowed to grow for three weeks. Mice were treated with placebo, AhR inhibitor CH223191 after castration surgery (n=5). *, $P < 0.05$, **, $P < 0.01$, ***, $P < 0.001$, and ****, $P < 0.0001$.

Supplementary Fig. S10

(a) Analysis of TDO2 mRNA levels in CRPC cell lines from single cell sequencing results (Data from Nature Cancer. 2022 Sep; 3(9):1071-1087). **(b-e)** The mRNA expression levels of *TDO2, AhR, CYP1A1* and *CYP1B1* in prostate cancer cell lines LNCaP, C4-2, 22RV1, DU145, PC3 and LNCaP-EnzR were measured by RT-qPCR. **(f)** RNA from LNCaP xenograft tumors was extracted, and mRNA expressions of *TDO2, AhR* and *CYP1A1* were detected by RT-qPCR (n=5). *, $P < 0.05$, **, $P < 0.01$, ***, $P < 0.001$, and ****, $P < 0.0001$.

Supplementary Fig. S11

(a) Use ImageJ to perform image inversion on Fig3H. **(b)** Repeat Fig3H and perform a quantitative analysis of the Western blot results using ImageJ. **(c)** The protein levels of TDO2 and AhR were measured in the PC3 cell line by knocking down TDO2 with shRNA, and the cell proliferation of PC3/Vec and PC3/shTDO2 cell lines was examined. **(d)** TDO2 was knocked down in the PC3 cells using shRNA, and the cells were examined for colony formation. **(e)** TDO2 was knocked down in the PC3 cells using shRNA, and cell migration was examined. **(f)** AhR was knocked down by siRNA in LNCaP-EnzR cells, the protein level of AhR was detected, and the cell proliferation was examined. **(g)** AhR was knocked down by siRNA in PC3 cells, the protein level of AhR was measured by Western blot, and the cell proliferation was examined. **(h)** CRPC cell line PC3 was treated with different concentrations of TDO2 inhibitor 680C91 and AhR inhibitor CH223191/BAY2416964 to examine their effects on cell growth. **(i)** The colony formation was examined for LNCaP-EnzR cells treated with TDO2 inhibitor 680C91 or AhR inhibitor CH223191/ BAY2416964. **(j)** PC3 line was treated with TDO2 inhibitor 680C91 and AhR inhibitor CH223191/BAY2416964 to detect the colony formation of the cells. *, $P < 0.05$, **, $P < 0.01$, ***, $P < 0.001$, and ****, $P < 0.0001$.

Supplementary Fig. S12

(a) The C4-2 cell line was treated with concentration gradient TDO2 inhibitor 680C91 and AhR inhibitor CH223191 to detect their effects on cell growth. **(b-c)** The C4-2 cell line was treated with TDO2 inhibitor 680C91 and AhR inhibitor CH223191 to detect their effects on cell cloning. **(d)**The 22RV1 cell line was treated with TDO2 inhibitor 680C91 and AhR inhibitor CH223191 in a concentration gradient to detect their effects on cell growth. **(e-f)** TDO2 inhibitor 680C91 and AhR inhibitor CH223191 were treated with 22RV1 cell line to detect their effects on cell cloning. **(g)** The DU145 cell line was treated with concentration gradient TDO2 inhibitor 680C91 and AhR inhibitor CH223191 to detect their effects on cell growth. **(h-i)** The DU145 cell line was treated with TDO2 inhibitor 680C91 and AhR inhibitor CH223191 to detect their effects on cell cloning. *, $P < 0.05$, **, $P < 0.01$, ***, $P < 0.001$, and ****, $P < 0.0001$.

Supplementary Fig. S13

(a) LNCaP-EnzR and PC3 treated with IDO1 inhibitor lindrodostat at different concentrations to examine the effects of lindrodostat on cell growth. **(b)** Cell proliferation was examined at days 0, 1, 3, 5 and 7 for PC3 cells treated with TDO2 inhibitor 680C91 or 680C91+Kyn. **(c)** Cell migration was examined at 0h, 24h and 48h for LNCaP-EnzR cells treated with TDO2 inhibitor 680C91 and 680C91+ Kyn. **(d)** Cell migration was examined at 0h, 24h and 48h for PC3 cells treated with TDO2 inhibitor 680C91 or 680C91+ Kyn. *, $P < 0.05$, **, $P < 0.01$, ***, $P < 0.001$, and ****, $P < 0.0001$.

Supplementary Fig. S14

(a) LNCaP-EnzR xenograft tumors were treated with placebo, TDO2 inhibitor LM10 or AhR inhibitor CH223191, and the tumor growth volume was recorded (n=6). **(b)** LNCaP-EnzR xenograft tumors were treated with placebo, TDO2 inhibitor LM10 or AhR inhibitor CH223191, and the tumor weight was recorded (n=6). **(c)** The LNCaP-EnzR xenograft tumors were treated separately with a placebo, TDO2 inhibitor LM10, the TDO2 inhibitor LM10+Kyn, AhR inhibitor CH223191, and the AhR inhibitor CH223191+Kyn. Tumor growth was monitored (n=4). **(d)** PC3 xenograft tumors were treated with placebo, TDO2 inhibitor LM10, and the tumor growth volume was recorded. **(e)** Extract proteins from LNCaP-EnzR tumors and detect the protein levels of AhR by western blot (n=6). **(f)** RNA from LNCaP-EnzR xenograft tumors was extracted, and mRNA expressions of *TDO2* and *AhR* were detected by RT-qPCR(n=6). *, $P < 0.05$, **, $P < 0.01$, ***, $P < 0.001$, and ****, $P < 0.0001$.

Supplementary Fig. S15

(a-c) The mRNA levels of *TDO2, AhR and CYP1A1* are detected using RT-qPCR in LNCaP cells with ADT treatment time gradients (0, 4, 7, 10, 14 days). **(d)** *TDO2* mRNA levels were measured in LNCaP cell lines treated with ADT for 7 days and 21 days. **(e-g)** The mRNA levels of *AR, FOXA1* and *GATA2* are detected using RT-qPCR in LNCaP cells with ADT treatment time gradients (0, 4, 7 days). **(h-i)** The LNCaP cells were treated with ADT, Enzalutamide, Bicalutamide, Apalutamide, and Darolutamide, and the mRNA levels of *AhR and CYP1A1* were detected using RT-qPCR. *, $P < 0.05$, **, $P < 0.01$, ***, $P < 0.001$, and ****, $P < 0.0001$.

Supplementary Fig. S16

(a-b) In the RNA sequencing database of 208 prostate cancer samples, the correlation between *TDO2* and *AR* and AR-related genes *SPDEF, FOXA1, CREB3L4, RAB6C, NKX3-1,* and *CCDC125* was analyzed. *, $P < 0.05$, **, $P < 0.01$, ***, $P < 0.001$, and ****, $P < 0.0001$.

Supplementary Fig. S17

(a) According to the base MOTIF of AR, the AR-regulated *TDO2* binding sequence was predicted on the eighth intron of *TDO2* (binding sites prediction website: https://jaspar.elixir.no/). **(b)** ChIP-seq data was used to verify the binding of AR as a transcription factor to *Tdo2* gene in VCAP -/+DHT cells. **(c)** ChIP-seq data showed the binding of AR as a transcription factor to *TDO2* gene in LNCaP, LNCaP/DHT, CW22RV1 and LNCaP95 cell lines. **(d)** Using the transcription factor prediction program (TRANSFAC version 8.3), 25 transcription factors (factors predicted within a dissimilarity margin less or equal than 1%) were found to bind to *TDO2* regulatory elements and have the potential to transcribe *TDO2*.

Supplementary Fig. S18

(a-b) The mRNA expression levels of *AR* and *GR* in prostate cancer cell lines LNCaP, C4-2, 22RV1, DU145, PC3 and LNCaP-EnzR were measured by RT-qPCR. **(c)** The protein of LNCaP xenograft tumor was extracted, and the expression of AR and GR was detected by western blot (n=5). **(d)** RNA was extracted from LNCaP tumors, and the mRNA levels of *AR* and *GR* were detected by RT-qPCR (n=5). **(e)** RNA was extracted from LNCaP tumors, and the mRNA levels of AR target genes *SLC2A3* and and GR target genes *KLK2* and *FKBP5* were detected by RT-qPCR (n=5). **(f)** The mRNA levels of *GR* and *AR* in LNCaP cell treated with ADT were measured. *, P < 0.05, **, P < 0.01, ***, P < 0.001, and ****, P < 0.0001.

Supplementary Fig. S19

(a-c) The GR antagonist Cort108297 or the GR agonist dexamethasone was used to treat LNCaP cells with or without ADT, and the mRNA levels of *TDO2, AhR,* and *CYP1A1* were detected using RT-qPCR. **(d-g)** GR is overexpressed in LNCaP cells, and the mRNA levels of *GR, TDO2, AhR,* and *CYP1A1* are detected by RT-qPCR with or without ADT. **(h)** Overexpress GR in LNCaP cells and examine the cell cycle of LNCaP under treatment with or without ADT. **(i)** GR was overexpressed in LNCaP cells, and the growth of LNCaP was tested under treatment with or without ADT. *, $P < 0.05$, **, $P < 0.01$, ***, $P < 0.001$, and ****, $P < 0.0001$.

Supplementary Fig. S20

(a) The GR antagonist Cort108297 or the GR agonist dexamethasone was used to treat LNCaP-EnzR cells with or without ADT, and the mRNA levels of *GR, TDO2, AhR,* and *CYP1A1* were detected using RT-qPCR. **(b)** LNCaP-EnzR cells are treated with a combination of GR antagonist Cort108297 and AhR inhibitor CH223191, and cell proliferation was tested. **(c)** GR was knocked down by siRNA in PC3 cells, the protein level of GR was measured by Western blot and the cell proliferation was examined. *, $P < 0.05$, **, $P < 0.01$, ***, $P < 0.001$, and ****, $P < 0.0001$.

Supplementary Fig. S21

(a) Transposon sequence (L1PA5), H3K27me3 methylation and GR binding site location in *TDO2* genome. **(b)** Pattern diagram of GR as a transcription factor replacing H3K27me3 to regulate *TDO2* transcription at L1PA5 after prostate cancer recurrence. **(c)** The L1 transposon subfamily L1PA5 has significant TF binding activity to GR (NR3C1).

Supplementary Fig. S22

(a) Perform image inversion on Fig.4N using ImageJ. **(b)** Repeat Fig.4N and perform a quantitative analysis of the Western blot results using ImageJ. **(c)** Perform image inversion on Fig.4O using ImageJ. **(d)** Repeat Fig.4O and perform a quantitative analysis of the Western blot results using ImageJ. *, $P < 0.05$, **, $P < 0.01$, ***, $P < 0.001$, and ****, $P < 0.0001$.

Supplementary Fig. S23

(a) Three regulatory sites of GR on the *TDO2* promoter. **(b)** GR and AR MOTIF (https://jaspar.elixir.no/). **(c)** The binding of GR to TDO2 transposable element was verified by ChIP-qPCR. *, $P < 0.05$, **, $P < 0.01$, ***, $P < 0.001$, and ****, $P < 0.0001$.

Supplementary Fig. S24

(a) KEGG enrichment analysis of 135 genes that were regulated by AhR as a transcription factor in all three conditions. **(b)** KEGG enrichment analysis in 274 genes specifically controlled by AhR in the early prostate cancer cell line LNCaP. **(c)** The CUT-Tag sequencing analysis was carried out on the transcription factors AhR. After normalization, LNCaP+ADT and LNCaP- EnzR groups are compared to achieve the intersection of genes with peaks greater than 100. **(d)** There were 247 overlapping genes between ADT group and EnzR group, and 112 genes were excluded from the 135 genes that were jointly regulated by the three groups. KEGG enrichment analysis was performed on these 112 genes. KEGG enrichment analysis tool: https://david.ncifcrf.gov/.

Supplementary Fig. S25

(a) Visualization diagram of *BCL6, SHC3* and *KRAS* genes in the three groups (using IGV analysis). **(b)** *AhR, CYP1A1, BCL6, SHC3* and *KRAS* mRNA levels were measured in LNCaP and LNCaP+ADT cells. **(c)** Visualization diagram of *CYP1A1*and *CYP1B1* genes in the three groups (using IGV analysis). **(d-e)** Visualization diagram of *CCND1, CREB3L2, NFKB1, CREB5, PPP2R3A, EDN1, HSPG2, REL,* and *ANAPC13* genes in the three groups (using IGV analysis). *, $P < 0.05$, **, $P < 0.01$, ***, $P < 0.001$, and ****, $P < 0.0001$.

Supplementary Table S1

Metabolites	Day14 (FBS)	Day14 (FBS)	Day14 (FBS)	Day14 (CCS)	Day14 (CCS)	Day14 (CCS)	Day14 (CCS)	Day14 (CCS)	HMDB	p-val	FC
Cystine	1000	1000	1000	1229426.049	865042.644	868410.675	614358.165	761524.17	HMDB00192	0.000685393	867.7523406
N1-(5-Phospho-D-ribosyl)glycinamide	1000	1000	1000	560907.993	512603.115	465067.185	299606.82	493111.305	HMDB02022	0.000227277	466.2592836
L-fucose/L-Fuculose	12411.18	19360.926	1000	273903.585	176189.586	342060.753	307303.932	249879.009	HMDB00174	0.000468259	24.70400038
L-cysteine	294258.81	230210.118	527428.752	4591980.666	3701002.575	3866650.584	4413795.798	3069778.899	HMDB00574	6.72291E-05	11.20444064
carnosine	640047.87	1813522.449	920201.958	8481020.103	8946882.138	8185956.744	2250766.128	9616181.139	HMDB00033	0.01212221	6.665679217
UDP-alpha-D-xylose	191646.828	213241.68	261114.894	1235929.068	879068.382	1069518.303	1544772.846	1121506.236	HMDB01018	0.000667322	5.270959413
indole-3-acetate/(5-hydroxyindol-3-yl)acetaldehyde	1292705.493	726871.107	1618144.47	5054626.512	4172767.155	5303838.735	9397017.876	4074768.501	HMDB00197	0.015870474	4.618773937
Formylkynurenine	188656.155	111830.16	278619.342	700621.095	690579.687	750566.544	873673.773	504980.664	HMDB01200	0.001062736	3.647439862
Hypotaurine	417531.231	449235.24	645252.654	1650597.123	1775014.323	1591746.774	1820976.435	1830105.213	HMDB00965	5.63756E-06	3.439813581
thiosulfate	118354.305	1502965.173	1172044.455	3708550.584	1701936.969	2934473.355	2659931.76	3183200.226	HMDB00257	0.012149259	3.047528335
(R)-mevalonate	7607725.44	8076348.573	8692118.562	21989297.83	21631195.76	23135431.18	24306769.47	21807834.98	HMDB59629	9.421E-07	2.778215561
3-Ureidoisobutyrate/Glycylsarcosine	1491678152	1485473095	1585852089	3958540393	3715800725	4025448855	4100860411	3815330090	HMDB02031	2.72541E-07	2.579351234
L-glutamine	1493241388	1487102844	1587200439	3958523720	3716363484	4026042250	4101222374	3815302768	HMDB00641	2.7328E-07	2.576980327
linoleic acid/linoelaidic acid (all trans C18:2)/octadecadienoate (n-C18:2)	17841521.95	19038464.25	18418356.62	49149768.33	29513570.18	41890155.96	46009469.85	41932076.11	HMDB00673	0.001973818	2.26222013
clupanodonic acid/docosa-4,7,10,13,16-pentaenoic acid	2302070.841	2067648.627	2560771.692	4368736.722	6557404.812	5782038.111	3959793.384	5282330.412	HMDB06528	0.003957433	2.246620291
spermidine dialdehyde-2	2573345.241	2279443.308	2426582.028	4772832.348	4615159.278	5251557.951	6015780.87	5026825.062	HMDB13076	0.000184247	2.116844189
arachidonic acid	18033900.77	15133906.7	19004459.02	29156931.9	43289173.13	40329908.02	26383814.87	35091302.41	HMDB01043	0.007056289	2.003951241
